# A Multiscale Quantitative Systems Pharmacology Model for the Development and Optimization of mRNA Vaccines

**DOI:** 10.1002/psp4.70041

**Published:** 2025-05-26

**Authors:** Lorenzo Dasti, Stefano Giampiccolo, Elisa Pettinà, Giada Fiandaca, Natascia Zangani, Lorena Leonardelli, Fabio De Lima Hedayioglu, Elio Campanile, Luca Marchetti

**Affiliations:** ^1^ Department of Cellular, Computational and Integrative Biology (CIBIO) University of Trento Trento Italy; ^2^ Fondazione the Microsoft Research—University of Trento Centre for Computational and Systems Biology (COSBI) Rovereto Italy; ^3^ Department of Information Engineering and Computer Science University of Trento Trento Italy; ^4^ School of Biosciences, Division of Natural Sciences University of Kent Canterbury UK; ^5^ Department of Mathematics University of Trento Trento Italy

## Abstract

The unprecedented effort to cope with the COVID‐19 pandemic has unlocked the potential of mRNA vaccines as a powerful technology, set to become increasingly pervasive in the years to come. As in other areas of drug development, mathematical modeling is a pivotal tool to support and expedite the mRNA vaccine development process. This study introduces a Quantitative Systems Pharmacology (QSP) model that captures key immune responses following mRNA vaccine administration, encompassing both tissue‐level and molecular‐level events. The model mechanistically describes the biological processes from the uptake of mRNA by antigen‐presenting cells at the injection site to the subsequent release of antibodies into the bloodstream. This two‐layer model represents a first attempt to link the molecular mechanisms leading to antigen expression with the immune response, paving the way for the future integration of specific vaccine attributes, such as mRNA sequence features and nanotechnology‐based delivery systems. Calibrated specifically for the BNT162b2 SARS‐CoV‐2 vaccine, the model has undergone successful validation across various dosing regimens and administration schedules. The results underscore the model's effectiveness in optimizing dosing strategies and highlighting critical differences in immune responses, particularly among low‐responder groups such as the elderly. Furthermore, the model's adaptability has been demonstrated through its calibration for other mRNA vaccines, such as the Moderna mRNA‐1273 vaccine, emphasizing its versatility and broad applicability in mRNA vaccine research and development.

**Summary**

- What is the current knowledge on the topic?
  ○ mRNA vaccines have emerged as a transformative technology in vaccinology, with mathematical modeling increasingly recognized as a key tool to optimize their development and enhance efficacy.
- What question did this study address?
  ○ This study introduces a novel multiscale Quantitative Systems Pharmacology (QSP) model that captures and predicts immune responses following mRNA vaccine administration, focusing on the integration of vaccine‐specific attributes.
- What does this study add to our knowledge?
  ○ The study provides a validated QSP model calibrated for BNT162b2 and adaptable to other mRNA vaccines, such as the Moderna mRNA‐1273 vaccine, offering mechanistic insights into dosing strategies and immune response variability in a population.
- How might this change drug discovery, development, and/or therapeutics?
  ○ The model serves as a versatile platform to accelerate mRNA vaccine design and optimization, improving dosing regimens and supporting the development of personalized vaccination strategies.

## 1 Introduction

During the SARS‐CoV‐2 pandemic, mRNA vaccines have emerged as an effective tool to counter and contain the virus spread [1]. Although this scenario was the first large‐scale test case for mRNA vaccine technology, research in this field spans more than three decades, focusing on employing mRNA vaccines as a tool to drive a potent adaptive immune response [2].

The need for a rapid response to the pandemic has highlighted the potential importance of computational models in supporting the design of optimal mRNA vaccine dosing and scheduling regimens [3]. The critical role of computational modeling in shortening the drug development process in other pharmaceutical areas has long been established [4]. Model‐informed drug development contributes to reducing time and costs, and regulatory agencies are currently evaluating the possibility of including *in silico* tools for Pre‐IND and IND submissions [5]. This could significantly improve vaccine development strategies that, historically, have predominantly relied on empirical approaches with minimal support from computational simulations.

The development of computational models for biological processes related to mRNA vaccines presents significant challenges due to the complexity and range of events these models must capture, from the translation of mRNA within host cells to the subsequent immune response culminating in antibody production [3]. The mechanistic modeling of antibody production began as early as 1970, with Bell (1970) [6] pioneering one of the first mathematical models. This foundational work has since been built upon, including notable contributions, such as the multiscale model from Chen et al. (2014) [7] for the immunogenicity of therapeutic proteins. In the aftermath of the COVID‐19 pandemic, the field has seen a surge in mathematical models specifically addressing the immune response to mRNA vaccines, with different levels of mechanistic detail. Korosec et al. (2022) [8] formulated a parsimonious model of the immune response to mRNA vaccines, establishing a baseline for further model refinement. Dogra et al. (2023) [9] proposed a detailed mechanistic model encompassing the respiratory tract, muscle, blood vessels, and lymphoid tissue, which directly represents the antigen at the injection site, albeit without explicitly modeling mRNA translation inside antigen‐presenting cells (APCs). Further extending the work of Chen et al. (2014) [7], Selvaggio et al. (2021) [10] focused on the early post‐administration events leading to the immune response in the draining lymph node. Voutouri et al. (2023) [11] introduced a more comprehensive model by extending previous work on COVID‐19 infection to include the immune response following vaccination. This model was the first to incorporate a phenomenological description of mRNA translation within APCs, representing a significant advancement in our understanding of mRNA vaccine‐induced immune responses. Ivaturi et al. (2024) [12] proposed an adaptation of the Immunostimulatory/Immunodynamics semi‐mechanistic framework [13], originally developed for traditional vaccines, to the mRNA vaccine context. In this model, mRNA translation into antigen and the processes leading to B‐cell activation are described phenomenologically. Similarly, Xu et al. (2024) [14] introduced a semi‐mechanistic mathematical platform to characterize the immune response to both traditional and mRNA vaccines.

**FIGURE 1:**
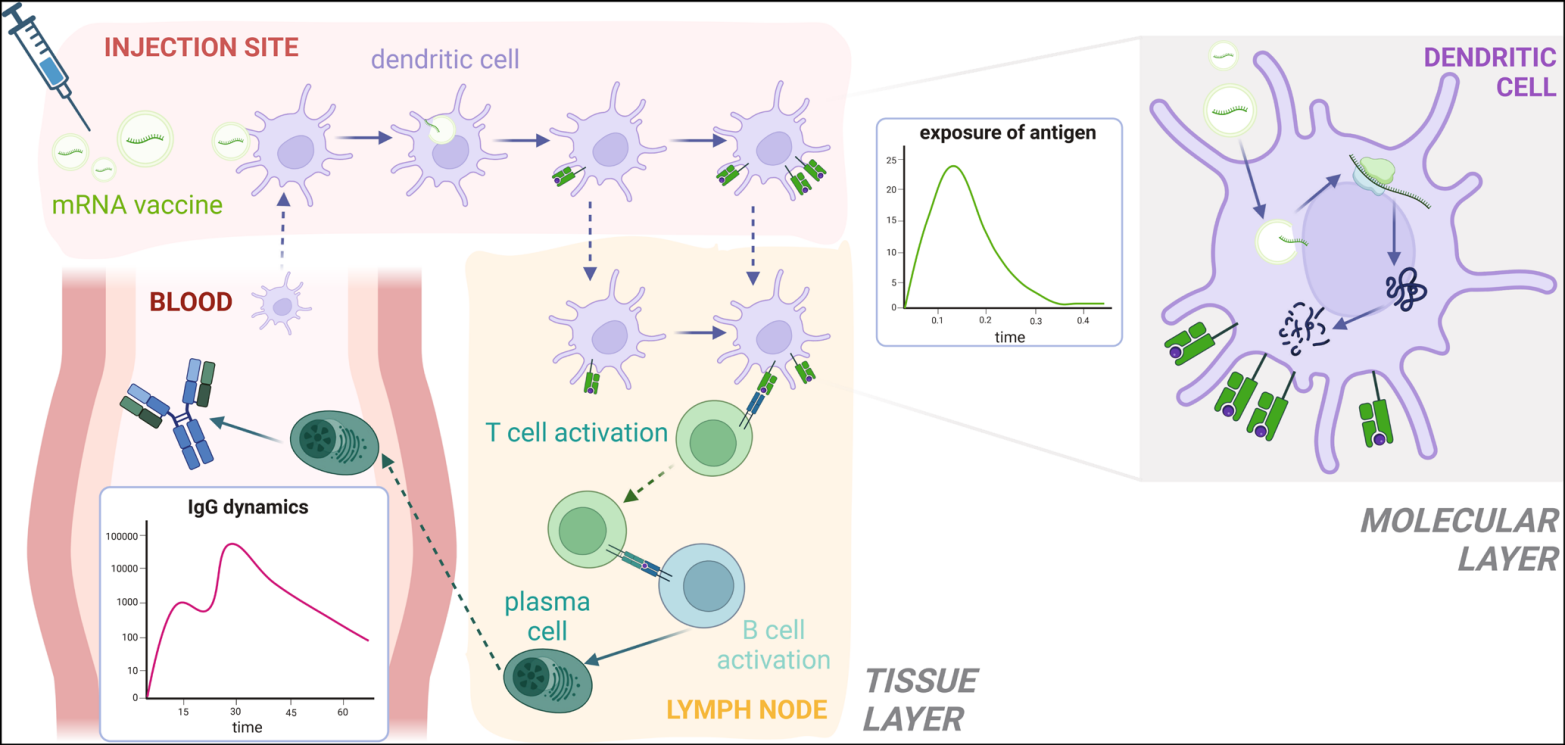
Model schematic. The tissue layer of the model (left), composed of the injection site, draining lymph node, and blood compartment, describes how the LNP‐encapsulated mRNA is uptaken, digested, and exposed by antigen‐presenting cells. This process is described in the molecular layer (right), which then informs the transition rate between different levels of antigen expression in the tissue layer of the model. Dendritic cells are responsible for the activation of T cells and, consequently, B cells: after the maturation process, plasma cells are produced, migrate to the bloodstream, and secrete antibodies. Dashed lines represent cell migration to different compartments; solid lines indicate a change in the state of a cell. Created in BioRender. Marchetti, L. (2025) https://BioRender.com/m06u736.

In this work, we propose a QSP model that extends the one introduced in Selvaggio et al. (2021) [10] and describes, with a higher level of mechanistic detail, the molecular and cellular events occurring after intramuscular (IM) injection of mRNA vaccines (Figure 1). Our model advances the existing literature by incorporating several key elements. Specifically, it introduces a molecular layer that describes the uptake of mRNA vaccines, their release within APCs, and the subsequent translation into vaccine antigens. The goal of this layer is to integrate molecular mechanisms into the description of the immune response, with the potential for these low‐level mechanisms to be directly linked to specific mRNA vaccine characteristics in the future, such as translation rate and the nanotechnology‐based delivery systems, thereby creating a platform that can be easily customized for different vaccines. Additionally, the model incorporates a tissue layer that captures regulatory events at the injection site, details the dynamics of antigen presentation among immune cells, and tracks B cell activity within the lymph node and bloodstream. These two layers are then integrated and synchronized into a unique multiscale model through the analysis of the exposure curve of MHCII‐antigen complexes on the surface of APCs.

The model was initially calibrated for the Pfizer‐BioNTech BNT162b2 vaccine and validated considering various dose regimens [15, 16, 17, 18, 19, 20, 21]. Our results show that the model accurately captures the antibody production for different administered doses and is able to identify *in silico* an optimal vaccine scheduling regimen able to guarantee continuous protection between subsequent vaccine administrations that is in agreement with the World Health Organization recommendations [22]. We then showcase how our mechanistic model can be applied to analyze differences in the biological processes underlying the immune response across a stratified population. Specifically, due to the significant impact of age on immune response following the administration of the BNT162b2 vaccine, we focused on an age‐stratified population. By calibrating the model with data from elderly individuals [16, 18, 23], we gained insights into age‐related variations in biological processes that aligned with existing literature. Finally, we employed literature data for the Moderna mRNA‐1273 vaccine to test the level of adaptability of our platform in describing other mRNA vaccines. Considering the parameter estimates previously computed for the BNT162b2 vaccine, data from the mRNA‐1273 vaccine (100 μg) [15] was enough to refine the parameter estimates and adapt the computational model, which was successfully validated on two other doses of the same product (25 μg and 50 μg) [24, 25], underscoring the versatility of our model and its broad applicability.

## 2 Materials and Methods

### 2.1 Experimental Data

Our model development was based on published data detailing the response of key model variables to mRNA vaccination. When available, data from human clinical studies were retrieved; otherwise, data from non‐human primates (NHPs) were employed. A comprehensive summary of the data used is provided in Table S7.

For calibrating the parameters describing the early events post‐vaccination at the injection site and draining lymph node, we relied on the data by Liang et al. (2017) [26], reporting the dynamics of different APC populations in rhesus macaques following the administration of an anti‐H10N8 influenza vaccine.

Parameters describing T cell, B cell, and antibody dynamics were calibrated using clinical data of the RBD‐binding IgG concentration in serum after vaccine administration. Various assays from different studies have been proposed to monitor vaccine‐induced antibody production, each employing its own units of measurement. Due to the challenges of directly comparing different units [27], we focused on data expressed in concentration (ng/mL) or comparable metrics. The normalization of the collected data was computed according to the methodology described in Supporting Information Section S4.

In particular, we employed the following studies for model calibration and validation:

- The BNT162b2 clinical trial [17], studies by Keshavarz et al. (2022) [15], Goel et al. (2021) [16], Kontopoulou et al. (2022) [18], Naaber et al. (2021) [19], Takeuchi et al. (2022) [20], and Payne et al. (2021) [21] to calibrate and validate the model for the BNT162b2 vaccine.
- Studies by Goel et al. (2021) [16], Kontopoulou et al. (2022) [18], and Röltgen et al. (2022) [23] to calibrate a version of the model using BNT162b2 data of over 60‐year‐old populations.
- The mRNA‐1273 vaccine clinical trial [24, 25] and the study by Keshavarz et al. (2022) [15] to calibrate and validate the model for the mRNA‐1273 vaccine.

The sample size of the experiments from which the experimental data are derived is reported in Table S8.

### 2.2 Parameter Calibration

Where possible, model parameter values were derived from existing literature. Otherwise, they were estimated by fitting the experimental data described in Section 3.1. To perform the fitting, a multistart optimization using non‐linear least squares was implemented [28], employing the *lsqnonlin* function in MATLAB. Given the multilayer and multiscale structure of our model, a stepwise approach was adopted for the calibration:

1. mRNA and APCs: initially, parameters related to mRNA and APCs were optimized using the data from Liang et al. (2017) [26]
2. Antigen presentation: the molecular layer was used to estimate the rates at which APCs change their level of antigen exposition.
3. T cells, B cells, and antibody production: the parameters related to the biological processes in the lymph nodes, which are directly linked to antibody production (such as T cell activation and B cell activation, proliferation, and differentiation), were calibrated using RBD IgG data.

A full description of the calibration procedure can be found in Supporting Information Section S5. Parameter bounds were set based on biological constraints from published studies, where available. Tables S3 and S6 provide detailed parameter estimates along with the data used for fitting. For parameters derived from literature, the source of the values is also included.

### 2.3 Uncertainty Quantification

The confidence intervals for the state of the dynamical system were computed following the approach used by Karelina et al. (2016) [29], which involves estimating the covariance matrix of the parameter estimates using the Fisher‐Information‐Matrix‐based method [30]. Assuming a multivariate normal distribution for the parameters, centered on the estimated values and with the estimated covariance matrix, we sampled 10,000 model parameterizations. These samples were then used to compute the nonparametric Monte Carlo confidence intervals [31] for the state of the dynamical system and for derived quantities.

### 2.4 Computational Environment

The model was implemented using *MATLAB 2023b*, and simulations were performed with the *ode15s* integrator. All equations are detailed in Supporting Information Section S1 and S2. The code to run the simulations presented in this paper is available at https://github.com/cosbi‐research/QSPmRNAVaccines.

## 3 Results

### 3.1 Model Definition

**FIGURE 2:**
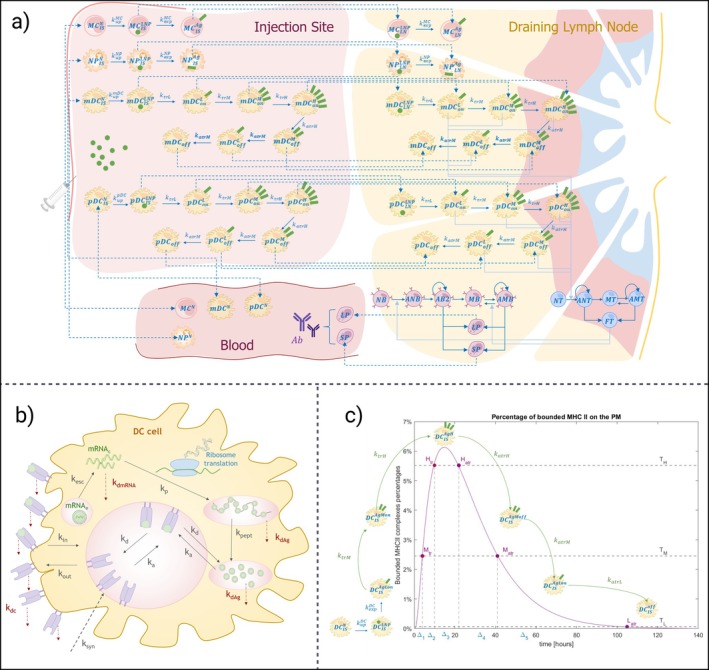
Model structure. (a) Tissue layer structure. The tissue layer is divided into three compartments: Injection site, draining lymph node, and blood. Four populations of APCs (monocytes, neutrophils, myeloid dendritic cells, and plasmacytoid dendritic cells) are modeled within both the injection site and the draining lymph node. In the lymph node compartment, various T and B lymphocyte populations are also represented. The blood compartment models plasma cells, antibodies, and the same four APC populations that are recruited at the injection site. Solid lines indicate cellular state transition or—where self‐pointed—cellular proliferation, dashed lines represent migration between compartments, and dotted lines depict activation of other cells. (b) Molecular layer structure. The molecular layer outlines key processes from the internalization of LNP‐encapsulated mRNA to antigen presentation via MHCII complexes on DCs. (c) Antigen exposition transition. The plot illustrates the method used to derive the transition rates between antigen exposure levels in dendritic cells based on a comparison between the MHCII‐antigen exposition curve (purple solid line) and the mean residence time of different populations of antigen‐expressing DCs. Additional details are available in Supporting Information Section S3. Created in BioRender. Marchetti, L. (2025) https://BioRender.com/r69t643.

The tissue layer consists of three different compartments: the injection site (modeled as muscle in the current version), a representative draining lymph node, and the bloodstream (Figure 2a). The molecular layer describes mRNA internalization, translation, and antigen presentation via MHCII complexes on the membrane of antigen‐presenting cells (Figure 2b) recruited in the injection site after the vaccine administration. These two layers are fully integrated into a unified platform, enhancing the model's descriptive potential for capturing key biological processes.

The tissue layer builds on a previously developed model by our group [10]. After vaccine administration at the injection site, the model describes the recruitment of various cell populations involved in the internalization of the vaccine particles, including monocytes, neutrophils, myeloid, and plasmacytoid dendritic cells (DCs). Simultaneously, it accounts for the degradation of mRNA by muscle cells or by other factors [32] activated when large doses are administered—a mechanism responsible for the non‐linear relationship between immune protection and dosage [33].

In our model, in accordance with recent studies [34], we assume that LNPs primarily distribute within the injected muscle and nearby lymph nodes, without entering systemic circulation. Additionally, we assume that only myeloid and plasmacytoid dendritic cells are responsible for the activation of T and B lymphocytes. Those populations of DCs, after the mRNA uptake, begin migrating toward the draining lymph nodes while simultaneously presenting the antigen via the MHCII complexes on their plasma membrane at different levels. The quantity of exposed antigens in the lymph nodes, along with the density of DCs and T cells, determines the activation of the latter. Once activated, T cells can either proliferate or differentiate into memory T cells or functional T cells. Memory T cells, upon subsequent exposure to the antigen, can be rapidly reactivated, while functional T cells are responsible for the activation of naïve B cells.

Following Bell (1970) [6], B cells are divided in our model into 17 distinct subgroups based on their affinity for the antigen. Upon activation, naïve B cells undergo an affinity maturation process in the germinal centers, where they proliferate and differentiate into either memory B cells or plasma cells (both short‐lived and long‐lived). Those processes of activation, proliferation, and differentiation are influenced by several factors, including the number of B cell receptors, the availability of antigen, the number of functional T cells, their carrying capacity, and the number of B cells available for activation. Memory B cells can be rapidly reactivated upon subsequent antigen exposure. Short‐ and long‐lived plasma cells migrate to the bloodstream, where they begin to produce antibodies.

The molecular layer, based on prior models [7, 35], captures the key biological processes occurring within APCs when they interact with LNP‐encapsulated mRNA. This includes mRNA internalization, endosomal escape, ribosomal translation, and the degradation of antigenic proteins into peptides, leading to the presentation of peptide‐MHCII complexes on APC membranes. This layer is designed to accommodate specific mRNA vaccine properties, such as mRNA translation rate and other dynamics that are influenced by mRNA encapsulation.

From the curve representing the number of peptide‐MHCII complexes present on the cell surface, the molecular layer informs the rates at which APCs alter their antigen exposure levels in the tissue layer (Figure 2c). The method used to derive these transition rates is detailed in Supporting Information Section S3.

### 3.2 Model Calibration

**FIGURE 3:**
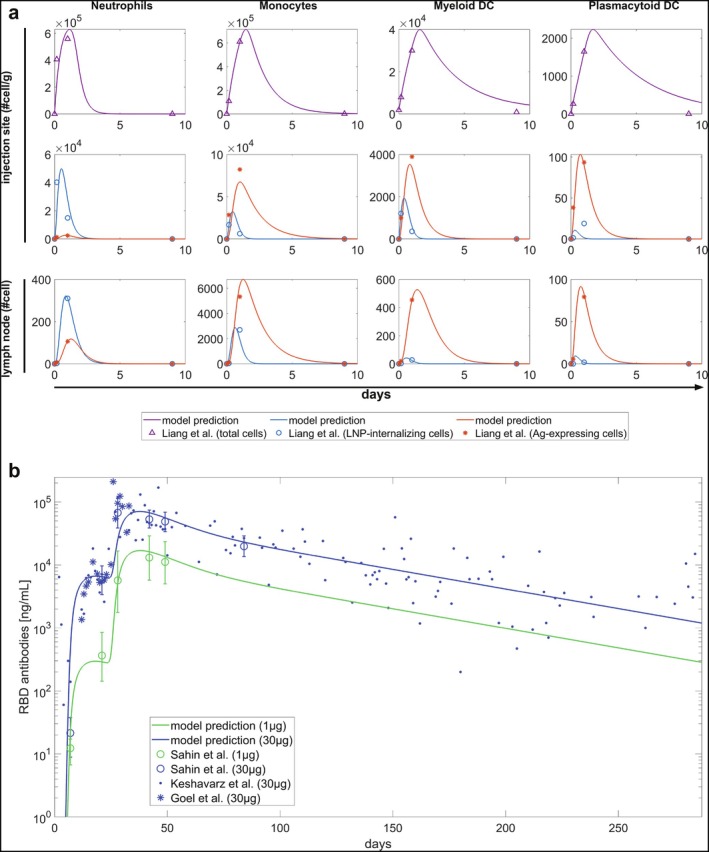
Model calibration. (a) Calibration using APC data. Dynamics of four different APC populations—one per column: neutrophils, monocytes, myeloid dendritic cells, and plasmacytoid dendritic cells. The first row of plots shows the total number of cells in each population at the injection site (purple triangles). The second row of plots depicts the dynamics of cells that have internalized the LNP‐coated mRNA (blue circles) and those expressing antigen on their surface (red asterisks). The third row shows the same populations as the second, but after they have migrated to the draining lymph node. Solid lines represent model predictions. (b) Calibration using BNT162b2 antibodies data. Dynamics of anti‐RBD IgG antibodies following the administration of 1 μg (green line) or 30 μg (blue line) of BNT162b2 vaccine. Data from Keshavarz et al. (2022) [15] and from Goel et al. (2021) [16] represent individual samplings, while the circles and error bars in the data from Sahin et al. (2021) [17] represent geometric mean concentrations with 95% confidence intervals. The y‐axis is shown on a logarithmic scale. Solid lines represent model predictions. Since Sahin et al. (2021) [17] data are expressed in different units, they have been multiplied by a factor derived as detailed in Supporting Information Section S4.

As outlined in Materials and Methods (Section 2), the model calibration involved several steps, with full details provided in Supporting Information Section S5. We calibrated the equations of the model related to APCs using data from Liang et al. (2019) [26], which included measurements of LNP uptake and antigen expression in monocytes, neutrophils, myeloid, and plasmacytoid DCs—both at the injection site and in the draining lymph node (Figure 3a)—after the mRNA vaccine administration. This allowed us to calibrate both global and population‐specific parameters for each of the four APC populations.

The maturation rates, defined as the progressive antigen presentation on mDC and pDC membranes, were estimated through the molecular layer, as described in Supporting Information Section S3 and S5.

For the model components involving T cells, B cells, and antibodies, we used serum concentration data for RBD IgG from Goel et al. (2021) [16] Keshavarz et al. (2022) [15] and Sahin et al. (2021) [17] (1 μg and 30 μg dose) following injection of the BNT162b2 (Figure 3b). The remaining rates were either set from literature or inferred from basic biological principles, such as maintaining a steady state in the absence of vaccination. A full list of calibrated parameters, along with those obtained from the literature, can be found in Table S3.

### 3.3 Model Validation

**FIGURE 4:**
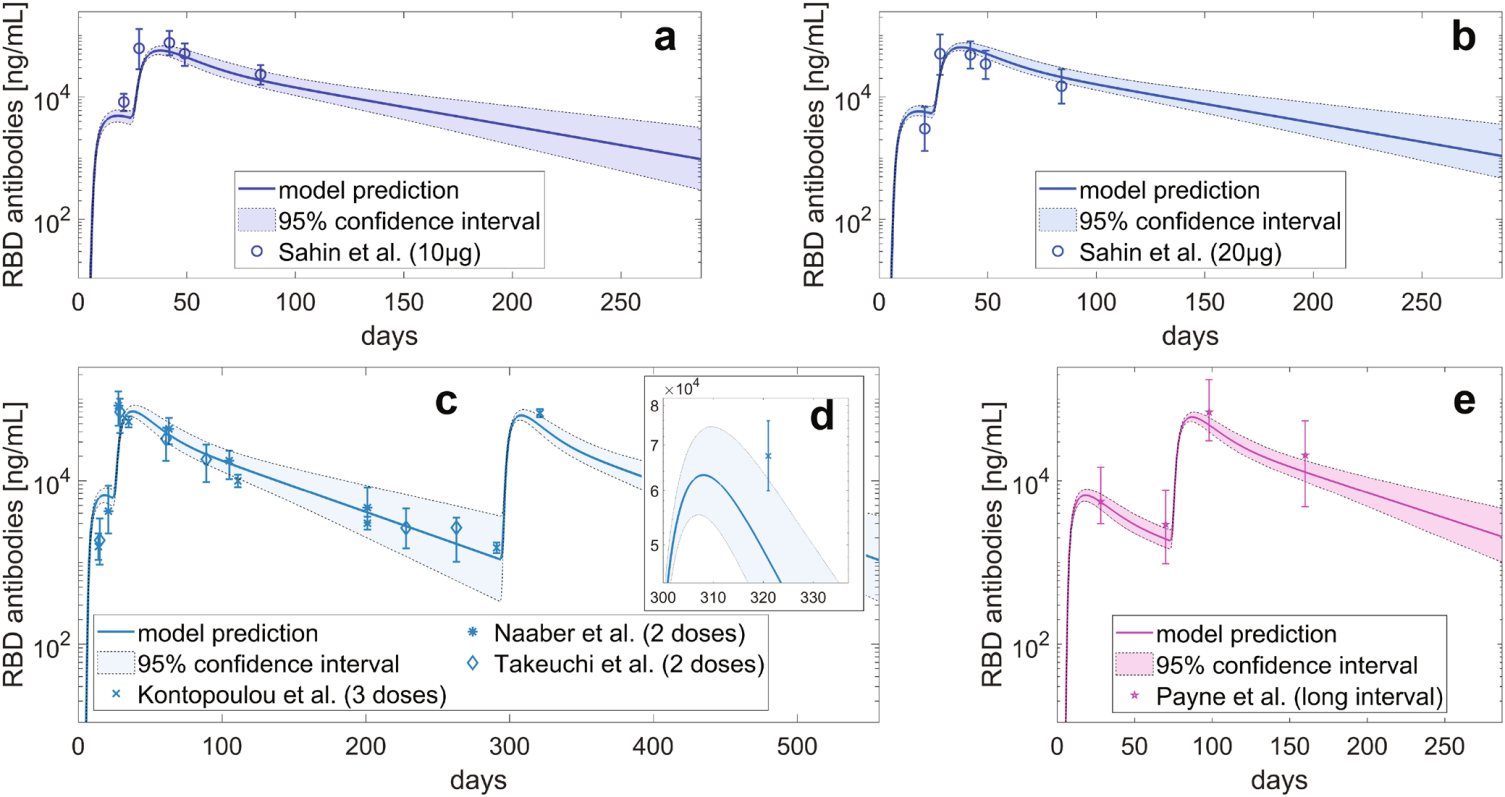
Model validation. (a) Dynamics of anti‐RBD IgG antibodies following the administration of two 10 μg doses of the BNT162b2 vaccine. (b) Dynamics of anti‐RBD IgG antibodies following the administration of two 20 μg doses of the BNT162b2 vaccine. (c) Dynamics of anti‐RBD IgG antibodies following the administration of three 30 μg doses of the BNT162b2 vaccine. (d) Zoom on the point of the panel (c) collected after the injection of a third dose of vaccine. (e) Dynamics of anti‐RBD IgG antibodies following the administration of two 30 μg doses of the BNT162b2 vaccine administered with an extended dosing interval (10 weeks). Solid lines represent model predictions, while shaded bands show the 95% confidence intervals. The y‐axes are on a logarithmic scale. Circles (Sahin et al. 2021) [17] with error bars indicate geometric mean concentrations with 95% confidence intervals; crosses (Kontopoulou et al. 2022) [18] with error bars represent geometric means with 95% confidence intervals; asterisks (Naaber et al. 2021) [19] with error bars show geometric mean concentrations with interquartile ranges; diamonds (Takeuchi et al. 2022) [20] with error bars represent geometric mean concentrations with interquartile ranges; stars (Payne et al. 2021) [21] with error bars represent median values with interquartile range. Due to differences in units of measurement, the plotted data were harmonized using scaling factors derived as explained in Supporting Information Section S4.

After fitting the model with BNT162b2 data, we performed three types of validation. First, we compared the model predictions with clinical trial data for BNT162b2, using two doses (10 μg and 20 μg) that were not included in the training set [17]. The 95% confidence intervals for the model variables are computed as described in Section 3.3. The model behavior demonstrated good agreement with the clinical data (Figure 4a,b).

Additionally, we incorporated two other datasets [18, 19] that followed the standard BNT162b2 protocol (two 30 μg doses administered 21 days apart), along with another dataset [20] based on a 3‐dose regimen, where the third dose was administered 9 months after the second. We compared these datasets with our model predictions, finding good agreement (Figure 4c) also in the time point following a third dose of the vaccine, where the 95% confidence interval positively intersects the error bars of the measurement—as shown in Figure 4d.

Finally, we successfully compared the model predicted behavior of the antibody curve with the data of the PITCH study provided in Payne et al. (2021) [21] (Figure 4e), where an extended dosing interval of 10 weeks between the first and second dose of BNT162b2 was taken into account.

### 3.4 Simulation of Optimal Dosing Schedules

Determining the optimal timing for subsequent vaccine administrations is a crucial aspect of vaccination strategy, particularly during a pandemic, as it can significantly influence the success or failure of a campaign. However, this is a challenging task that can be approached from different perspectives and where many aspects are involved. In this study, we focused on the aim of ensuring continuity in protection by trying to avoid lapses that can lead to breakthrough infections between the first and the second vaccine dose. To this aim, we used our model to simulate various *in silico* scenarios by varying the timing of the second dose of the BNT162b2 vaccine—ranging from 14 to 54 days after the initial injection. Over a 160‐day period, we compared the model‐predicted levels of antibodies with a literature‐based protection threshold against SARS‐CoV‐2, derived from Giorgi et al. (2021) [36].

**FIGURE 5:**
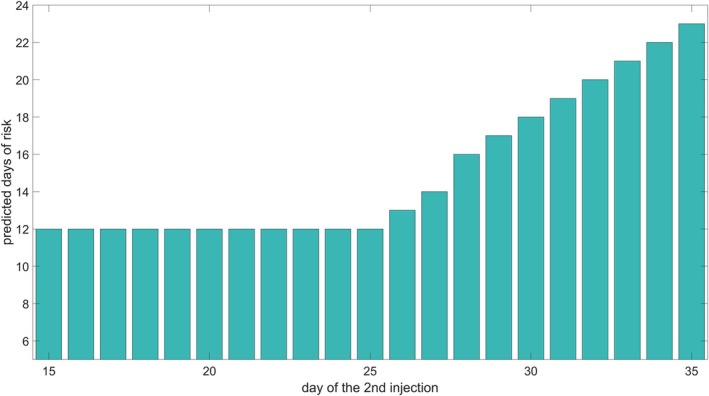
Model predicted number of days at risk. The plot shows the predicted number of days at‐risk (*y*‐axes), during which the 95% confidence interval for the model predicted anti‐RBD IgG antibodies in serum is not completely above the protective threshold, based on the timing of the second dose administration (*x*‐axis). The protective antibody level, derived from the literature [36], is 4422.11 ng/mL.

We analyzed the 95% confidence intervals of the model predicted anti‐RBD IgG antibody levels over time, computed as described in Section 3.3. These intervals were compared to the protective threshold established by Giorgi et al. (2021) [36], defined as the geometric mean concentration of anti‐RBD IgG in convalescent serum (4422.11 ng/mL), which represents the antibody concentration considered protective. The days when the confidence intervals of the anti‐RBD IgG antibody levels were fully above the protective threshold were classified as protected days, while the remaining ones were considered at‐risk. We then assessed the number of at‐risk days for various simulated schedules of the second vaccine dose (Figure 5).

The results indicate that when the second dose is administered later than 14 days post‐injection, the number of at‐risk days predicted by the model does not immediately increase but remains constant for a certain period. This constant value corresponds to the time required for antibodies to appear in the serum and reach a protective threshold after the first injection (approximately 12 days). In other words, the model suggests that for schedules within the flat region of the plot, antibody levels (at a 95% confidence level) ensure continuous protection throughout the simulation period, without any lapses in protection. Adopting the practice of maximizing the interval between two vaccine administrations to reduce potential adverse events, our model‐based approach suggests an optimal timing for the second dose around day 25 post‐first injection, aligning with the vaccine manufacturer's recommendations and the WHO initial interim guidance (2021) [22]. Different findings are reported in the literature regarding optimal dosing intervals. Payne et al. (2021) [21] observed a higher mean serological response when the second dose was administered 10 weeks after priming, leading to the conclusion that an extended interval is preferable. This finding is not inconsistent with our model predictions since, as shown in Figure 4e, our simulations have been successfully compared with the experimental data from Payne et al. (2021) [21]. However, plotting the data from Payne et al. (2021) [21] against the protective threshold from Giorgi et al. (2021) [36] reveals that the antibody levels before a second dose administered 10 weeks after priming fall below the protective threshold (see Figure S2). This suggests that, in terms of continuity of protection, a shorter interval between the two doses may be preferred.

### 3.5 Stratified Data and Related Biological Aspects

The COVID‐19 pandemic and its associated vaccination campaigns have underscored the importance of understanding immune response variation across different population segments, enabling more effective and tailored vaccination strategies [37]. QSP models, which encode complex biological processes, are powerful tools for enhancing our understanding of the key biological factors driving these immune responses. With this approach, we sought to calibrate our model using stratified data, emphasizing the differences in parameter estimates to uncover biological insights.

**FIGURE 6:**
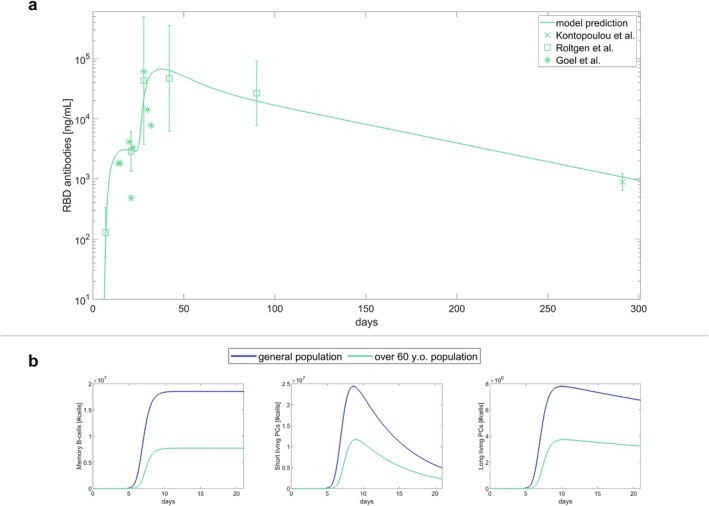
Model calibration using BNT162b2 antibody data for the elderly population and comparison with the general population. (a) Dynamics of anti‐RBD IgG antibodies following the administration of 30 μg of the BNT162b2 vaccine in individuals aged over 60. Data from Goel et al. (2021) [16] represent individual samples, while crosses from Kontopoulou et al. (2022) [18] and squares from Röltgen et al. (2022) [23] with error bars represent geometric mean concentrations with 95% confidence intervals. The y‐axis is in log scale, and the solid line shows the model prediction. Since the data from Röltgen et al. (2022) [23] and Kontopoulou et al. (2022) [18] are expressed in different units, they have been scaled by a factor deduced as explained in Supporting Information Section S4. (b) Comparison of the model dynamics between the general population (dark blue line) and the over‐60 population (light blue line) for memory B cells in the lymph node (left panel), short‐lived plasma cells in the blood (central panel) and long‐lived plasma cells in the blood (right panel). The dynamics cover the period of 21 days between the first and the second dose administration.

We gathered antibody data for the BNT162b2 vaccine from individuals over 60 years old [15, 24, 25]. In the calibration process, we assumed that the dynamics of APCs in older adults remained largely unchanged compared to the general population. Instead, we hypothesized that the primary differences arise in the immune response within lymph nodes. Thus, to fit the stratified data, we reoptimized the model parameters related to T cells, B cells, and antibodies (Figure 6a).

Leveraging the model, we compared the immune dynamics generated from this new parameter set with those derived from the general population data. In Figure 6b, we illustrate these comparisons for B cell phenotypes, where we observed the greatest differences: memory B cells, short‐lived plasma cells, and long‐lived plasma cells. The plots cover the time frame between the first and second immunization, as this is when the most significant differences in our simulations occur. A slower and weaker dynamic is clearly observed in the older population compared to the general one.

Finally, we calculated the relative differences between these new parameter estimates and those obtained from the general population. Further details about the optimization procedure and parameter values can be found in Supporting Information Section S5 and Table S9. The most substantial difference was observed in the parameter governing B cell maturation in germinal centers, with a significantly lower value indicating a slower maturation process in older individuals. This age‐related decline in the number and efficacy of germinal centers is well‐supported in the literature [38, 39]. We also noted smaller yet significant decreases in the proliferation rate of memory B cells and in the migration rate of plasma cells from the lymph nodes to the bloodstream.

### 3.6 Calibration With Data of Different Vaccines and Insights on Optimal Dose

**FIGURE 7:**
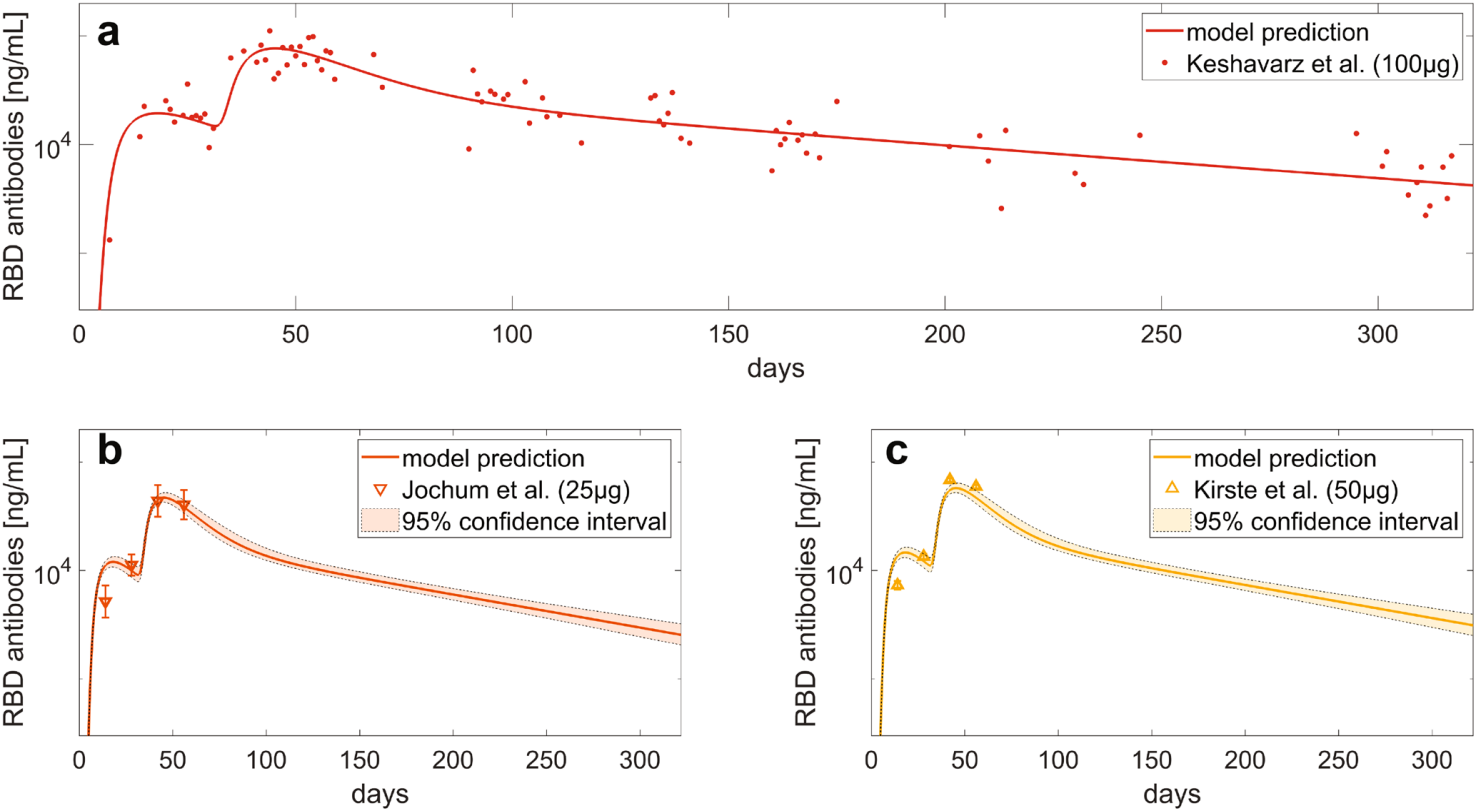
Model calibration and validation using mRNA‐1273 antibody data. (a) Model calibration: dynamics of anti‐RBD IgG antibodies following the administration of 100 μg of mRNA‐1273 vaccine. (b, c) Model validation: dynamics of anti‐RBD IgG antibodies following the administration of 25 μg (b) or 50 μg (c) doses of the mRNA‐1273 vaccine. Error bars in the data from Jochum et al. (2022) [24] and Kirste et al. (2023) [25] represent geometric mean concentrations with 95% confidence intervals. The y‐axes are in log scale. Solid lines indicate model predictions. To align different units of measurement, data have been rescaled as described in Supporting Information Section S4.

An additional objective of our study was to test how well our model, once calibrated with data from one vaccine, could be adapted to other vaccine products while retaining previously derived insights. For this, we collected data on the mRNA‐1273 vaccine at a 100 μg dose [15] and attempted to re‐fit the model to these data (Figure 7a), using the parameter set obtained for the BNT162b2 as a starting point. In doing so, we assumed that the events occurring at the injection site would remain unchanged. Details of the optimized parameters can be found in Supporting Information Section S5 and in Table S9. For further validation, we incorporated data from two additional dose levels obtained from Phase 1 [24] and Phase 2 [25] clinical trials of the mRNA‐1273 vaccine. This test was performed to assess the degree of adaptability of our model in being repurposed for a novel vaccine product by leveraging a minimum amount of data. In our tests, with the aid of our model, data from a single dose could be sufficient to adapt the model and explore *in silico* alternative dosing protocols. The validation results (Figure 7b,c) suggest that the model successfully captured the general behavior of the antibody dynamics—particularly after the first weeks post‐priming—and accurately reflected the changes in response to different dose levels.

## 4 Discussion

In this study, we present a mechanistic model that provides a comprehensive, multi‐layered description of the biological processes following mRNA vaccine administration, up to antibody release in the bloodstream. Expanding on the work of Selvaggio et al. (2021) [10], we incorporated the mRNA translation within APCs (molecular layer), the blood compartment, and a detailed representation of B cell affinity maturation and mRNA degradation. The model was calibrated for the BNT162b2 vaccine, using clinical data when available, supplemented with nonhuman primate data when strictly necessary.

The validation results reported in Figure 4 demonstrate the model's ability to generalize across diverse dose regimens and vaccine administration schedules. The model also proved suitable for identifying an optimal vaccination schedule that ensures continuity of protection between doses, aligning with manufacturer guidelines and the World Health Organization recommendations [22]. Additionally, the model identifies key immune factors contributing to the diminished antibody response in elderly individuals, aligning with existing literature. Finally, Figure 7 shows that the model can be repurposed for a novel vaccine product, specifically the mRNA‐1273 vaccine. These findings underscore the model's potential as a foundational mechanistic platform for supporting mRNA vaccine development.

Our work has some limitations. The current version of the model focuses on FDA‐approved mRNA vaccines against COVID‐19, due to their importance in responding to the last pandemic, and it has been calibrated for the mRNA‐1273 and BNT162b2 vaccines. Although we explicitly described the translation process to directly incorporate the translation rate of an mRNA sequence into our model, we have not yet incorporated data from other mRNA vaccines, which may have different RNA translation rates. Additionally, some vaccine attributes—such as the composition of the LNP delivery system—have not yet been mapped to specific molecular‐level parameters and remain opportunities for further refinement. Further investigation is needed to strengthen the platform versatility across different mRNA vaccines (see Supporting Information Section S7.1 for a more detailed discussion).

The complexity of our mechanistic description of the immune response, despite being a step toward a complete mechanistic approach to modeling the immune response of mRNA vaccines, presents significant challenges. This complexity requires extensive experimental data for calibration while simultaneously challenging parameter identifiability due to the large number of variables and equations. This contrasts with the reduced‐complexity description offered by semi‐mechanistic approaches, such as Immunostimulatory/Immunodynamic (IS/ID) models [12]. However, the data‐driven components of these models require vaccine‐specific calibrations, which limit their ability to predict outcomes in unforeseen scenarios—such as changes in vaccine characteristics (see Supporting Information Section S7.2 for a more detailed comparison between our approach and IS/ID models).

To ensure model parsimony, certain biological processes were omitted. The MHC II complex is represented phenomenologically without differentiating between human variants. While incorporating these aspects could enhance personalization through host immunogenomics, our study focuses on the average humoral response to an mRNA vaccine. Including such details would introduce complexities beyond the scope of this work. Additionally, some processes were excluded due to a lack of clear consensus in the literature. Specifically, the potential systemic circulation of the translated antigen—reported inconsistently across studies [40, 41, 42]—was not considered. Similarly, immune imprinting, the tendency of the immune system to rely on pre‐existing memory when encountering related variants [43], was excluded due to the uncertainty surrounding its precise effects [44, 45], making its integration into a mechanistic framework challenging. We also did not address the *in silico* evaluation of antibody binding affinity loss due to changes in spike protein epitope topology. Further discussion of the limitations of our model in predicting immune responses to viral variants is provided in Supporting Information Section S7.3.

Future work can expand our model in several directions. One potential extension is incorporating reactogenicity, a key aspect of vaccination. Additionally, we aim to enhance the platform's versatility by integrating data from various mRNA vaccines [46, 47, 48, 49]. Leveraging state‐of‐the‐art techniques, the platform can be calibrated to quantify biological variability that drives heterogeneity in immune responses to mRNA vaccines. As part of this effort, the MHC II complex could be further detailed to account for the host HLA genotype, a crucial factor influencing individual immune responses [50]. Finally, future extensions may include modeling alternative injection sites, depending on data availability, and adapting the model for preclinical applications.

## Author Contributions

All authors wrote the manuscript. L.D., S.G., G.F., N.Z., L.L., and L.M. designed the research. L.D., S.G., E.P., G.F., N.Z., L.L., F.D.L.H., E.C., and L.M. performed the research and analyzed the data.

## Conflicts of Interest

The authors declare no conflicts of interest.

## Supporting information


File S1.
