# Supplementary material for: A Multiscale Quantitative Systems Pharmacology Model for the Development and Optimization of mRNA Vaccines
Source: CPT Pharmacometrics Syst Pharmacol. 2025 May 26;14(7):1213–24. doi: 10.1002/psp4.70041 (PMC12256567; doi:10.1002/psp4.70041)
Supplement: Supplementary file 1 — File S1. [file PSP4-14-1213-s001.pdf]

A multiscale Quantitative Systems Pharmacology model for the development and optimization of mRNA vaccines

Lorenzo Dasti, Stefano Giampiccolo, Elisa Pettinà, Giada Fiandaca, Natascia Zangani, Lorena Leonardelli, Fabio De Lima Hedayioglu, Elio Campanile, Luca Marchetti

Supplementary File S1

SUMMARY

S1. QSP tissue layer for mRNA vaccines 2
S1.1 Model variables 2
S1.2 Initial values 4
S1.3 Model parameters 6
S1.4 Model equations 11
S1.4.1 Injection Site – IS 11
S1.4.2 Draining Lymph Node - LN 14
S1.4.3 Blood – BL 22
S2. Molecular layer for mRNA vaccines 23
S2.1 Model variables 23
S2.2 Initial values 23
S2.3 Model parameters 24
S2.4 Model equations 24
S3. Molecular and tissue layers connection 26
S4. Antibody level experimental data and harmonization of different measure units 27
S5. Calibration procedure 30
S6. Extended dosing interval of BNT162b2 vaccine 31
S7. Supplementary Discussion 32
S7.1 Model extension to other vaccines 32
S7.2 Novelty against existing IS/ID models 32
S7.3 Model capability to describe the immune response to virus mutation 33
Bibliography 34

S1. QSP TISSUE LAYER FOR MRNA VACCINES

We provide a detailed account of the tissue layer, including tables listing the model variables (**Section S1.1**), initial conditions (**Section S1.2**), and parameter estimates (**Section S1.3**), categorized by their respective biological compartments. Additionally, **Section S1.4** contains a comprehensive description of all the equations pertaining to this layer.

S1.1 MODEL VARIABLES

Variable name	Variable description	Units
	**Injection Site (IS)**	
$\mathbf{mRNA_{IS}}$	mRNA product	pmol
$\mathbf{NP_{IS}}$	Resident naïve neutrophils (NPs) at IS	#cells
$\mathbf{NP_{IS}^{LNP}}$	NPs that internalized the LNPs at IS	#cells
$\mathbf{NP_{IS}^{Ag}}$	NPs expressing the antigen protein at IS	#cells
$\mathbf{MN_{IS}}$	Resident naïve monocytes (MNs) at IS	#cells
$\mathbf{MN_{IS}^{LNP}}$	MNs that internalized the LNPs at IS	#cells
$\mathbf{MN_{IS}^{Ag}}$	MNs expressing the antigen protein at IS	#cells
$\mathbf{mDC_{IS}}$	Resident naïve myeloid dendritic cells (mDCs) at IS	#cells
$\mathbf{mDC_{IS}^{LNP}}$	mDCs that internalized the LNPs at IS	#cells
$\mathbf{mDC_{IS}^{Ag}L_{on}}$	mDCs with low level of antigen expression at IS (binding-phase)	#cells
$\mathbf{mDC_{IS}^{Ag}M_{on}}$	mDCs with medium level of antigen expression at IS (binding-phase)	#cells
$\mathbf{mDC_{IS}^{Ag}H}$	mDCs with high level of antigen expression at high level at IS	#cells
$\mathbf{mDC_{IS}^{Ag}M_{off}}$	mDCs with medium level of antigen expression at IS (unbinding-phase)	#cells
$\mathbf{mDC_{IS}^{Ag}L_{off}}$	mDCs with low level of antigen expression at IS (unbinding-phase)	#cells
$\mathbf{mDC_{IS}^{off}}$	Mature mDCs with no antigen expression at IS (unbinding-phase endpoint)	#cells
$\mathbf{pDC_{IS}}$	Resident naïve plasmacytoid dendritic cells (pDCs) at IS	#cells
$\mathbf{pDC_{IS}^{LNP}}$	pDCs that internalized the LNPs at IS	#cells
$\mathbf{pDC_{IS}^{Ag}L_{on}}$	pDCs with low level of antigen expression at IS (binding-phase)	#cells
$\mathbf{pDC_{IS}^{Ag}M_{on}}$	pDCs with medium level of antigen expression at IS (binding-phase)	#cells
$\mathbf{pDC_{IS}^{Ag}H}$	pDCs with high level of antigen expression at IS	#cells
$\mathbf{pDC_{IS}^{Ag}M_{off}}$	pDCs with medium level of antigen expression at IS (unbinding-phase)	#cells
$\mathbf{pDC_{IS}^{Ag}L_{off}}$	pDCs with low level of antigen expression at IS (unbinding-phase)	#cells
$\mathbf{pDC_{IS}^{off}}$	Mature pDCs with no antigen expression at IS (unbinding-phase endpoint)	#cells
	**Draining Lymph Node (LN)**	
$\mathbf{NP_{LN}^{LNP}}$	NPs that internalized the LNPs at LN	#cells
$\mathbf{NP_{LN}^{Ag}}$	NPs in which the mRNA is translated, leading to antigen protein production at LN	#cells
$\mathbf{MN_{LN}^{LNP}}$	MNs that internalized the LNPs at LN	#cells

$\mathbf{MN_{LN}^{Ag}}$	MNs in which the mRNA is translated, leading to antigen protein production at LN	#cells
$\mathbf{mDC_{LN}^{LNP}}$	mDCs that internalized the LNPs at LN	#cells
$\mathbf{mDC_{LN}^{Ag}L_{on}}$	mDCs with low level of antigen expression at LN (binding-phase)	#cells
$\mathbf{mDC_{LN}^{Ag}M_{on}}$	mDCs with medium level of antigen expression at LN (binding-phase)	#cells
$\mathbf{mDC_{LN}^{Ag}H}$	mDCs with high level of antigen expression at LN	#cells
$\mathbf{mDC_{LN}^{Ag}M_{off}}$	mDCs with medium level of antigen expression at LN (unbinding-phase)	#cells
$\mathbf{mDC_{LN}^{Ag}L_{off}}$	mDCs with low level of antigen expression at LN (unbinding-phase)	#cells
$\mathbf{mDC_{LN}^{off}}$	Mature mDCs with no antigen expression at LN (unbinding-phase endpoint)	#cells
$\mathbf{pDC_{LN}^{LNP}}$	pDCs that internalized the LNPs at LN	#cells
$\mathbf{pDC_{LN}^{Ag}L_{on}}$	pDCs with low level of antigen expression at LN (binding-phase)	#cells
$\mathbf{pDC_{LN}^{Ag}M_{on}}$	pDCs with medium level of antigen expression at LN (binding-phase)	#cells
$\mathbf{pDC_{LN}^{Ag}H}$	pDCs with high level of antigen expression at LN	#cells
$\mathbf{pDC_{LN}^{Ag}M_{off}}$	pDCs with medium level of antigen expression at LN (unbinding-phase)	#cells
$\mathbf{pDC_{LN}^{Ag}L_{off}}$	pDCs with low level of antigen expression at LN (unbinding-phase)	#cells
$\mathbf{pDC_{LN}^{off}}$	Mature pDCs with no antigen expression at LN (unbinding-phase endpoint)	#cells
$\mathbf{NT}$	Naïve T-helper (Th) cells at LN	#cells
$\mathbf{AT_N}$	Activated Th cells derived from naïve T-cells at LN	#cells
$\boldsymbol{MT}$	Memory T-cells at LN	#cells
$\boldsymbol{AT_M}$	Activated Th cells derived from memory T-cells at LN	#cells
$\boldsymbol{FT}$	Functional T-cells at LN	#cells
$\boldsymbol{NB}$	Naïve B-cells at LN	#cells
$\boldsymbol{ANB}$	Activated B-cells derived from naïve B-cells at LN	#cells
$\boldsymbol{GCB}$	Activated B-cells that entered the germinal center	#cells
$\boldsymbol{MB}$	Memory B-cells at LN	#cells
$\boldsymbol{AMB}$	Activated B-cells derived from memory B-cells at LN	#cells
$\boldsymbol{SP}$	Short-lived plasma (antibody secreting) B-cells	#cells
$\boldsymbol{LP}$	Long-lived plasma (antibody secreting) B-cells	#cells
**Blood (BL)**		
$\boldsymbol{NP_{BL}}$	Naïve NPs in BL	#cells
$\boldsymbol{MN_{BL}}$	Naïve MNs in BL	#cells
$\boldsymbol{mDC_{BL}}$	Naïve mDCs in BL	#cells
$\boldsymbol{pDC_{BL}}$	Naïve pDCs in BL	#cells
$\boldsymbol{SP_{BL}}$	Short-lived plasma (antibody secreting) B-cells in BL	#cells
$\boldsymbol{LP_{BL}}$	Long-lived plasma (antibody secreting) B-cells in BL	#cells
$\boldsymbol{Ab_{BL}}$	Anti-spike IgG antibodies in BL	pmol

Table S1: *model variables of the tissue layer.*

S1.2 Initial values

Parameter	Description	Initial value	Units	Reference
	**Injection Site (IS)**			
$mRNA0_{IS}$	Initial amount of mRNA at IS	21.7789	pmol	[1]
$NP0_{IS}$	Initial number of naïve NPs at IS	0	#cells	[2]
$NP0_{IS}^{LNP}$	Initial number of NPs that internalized the LNPs at IS	0	#cells	-
$NP0_{IS}^{Ag}$	Initial number of NPs expressing antigen at IS	0	#cells	-
$MN0_{IS}$	Initial number of naïve MNs at IS	300	#cells	[2]
$MN0_{IS}^{LNP}$	Initial number of MNs that internalized the LNPs at IS	0	#cells	-
$MN0_{IS}^{Ag}$	Initial number of MNs expressing antigen at IS	0	#cells	-
$mDC0_{IS}$	Initial number of naïve mDCs at IS	1.8644e+03	#cells	[2]
$mDC0_{IS}^{LNP}$	Initial number of mDCs that internalized the LNPs at IS	0	#cells	-
$mDC0_{IS}^{Ag}L_{on}$	Initial number of mDCs with low antigen expression at IS (binding-phase)	0	#cells	-
$mDC0_{IS}^{Ag}M_{on}$	Initial number of mDCs with medium antigen expression at IS (binding-phase)	0	#cells	-
$mDC0_{IS}^{Ag}H$	Initial number of mDCs with high antigen expression at IS	0	#cells	-
$mDC0_{IS}^{Ag}M_{off}$	Initial number of mDCs with medium antigen expression at IS (unbinding-phase)	0	#cells	-
$mDC0_{IS}^{Ag}L_{off}$	Initial number of pDCs with low antigen expression at IS (unbinding-phase)	0	#cells	-
$mDC0_{IS}^{off}$	Initial number of mature mDCs with no antigen expression at IS	0	#cells	-
$pDC0_{IS}$	Initial number of naïve pDCs at IS	0	#cells	[2]
pDC_{IS}^{LNP}	Initial number of pDCs that internalized the LNPs at IS	0	#cells	-
$pDC_{IS}^{Ag}L_{on}$	Initial number of pDCs with low antigen expression at IS (binding-phase)	0	#cells	-
$pDC_{IS}^{Ag}M_{on}$	Initial number of pDCs with medium antigen expression at IS (binding-phase)	0	#cells	-
$pDC_{IS}^{Ag}H$	Initial number of pDCs with high antigen expression at IS	0	#cells	-
$pDC_{IS}^{Ag}M_{off}$	Initial number of pDCs with medium antigen expression at IS (unbinding-phase)	0	#cells	-
$pDC_{IS}^{Ag}L_{off}$	Initial number of pDCs with low antigen expression at IS (unbinding-phase)	0	#cells	-
pDC_{IS}^{off}	Initial number of mature pDCs with no antigen expression at IS	0	#cells	-
	**Draining Lymph Node (LN)**			
$NP0_{LN}^{LNP}$	Initial number of NPs that internalized the LNPs in LN	0	#cells	-
$NP0_{LN}^{Ag}$	Initial number of NPs with expressed antigen in LN	0	#cells	-
$MN0_{LN}^{LNP}$	Initial number of MNs that internalized the LNPs in LN	0	#cells	-

$\boldsymbol{MN0_{LN}^{Ag}}$	Initial number of MNs with expressed antigen in LN	0	#cells	-
$\boldsymbol{mDC0_{LN}^{LNP}}$	Initial number of mDCs that internalized the LNPs in LN	0	#cells	-
$\boldsymbol{mDC0_{LN}^{Ag}L_{on}}$	Initial number of mDCs with low antigen expression in LN (binding-phase)	0	#cells	-
$\boldsymbol{mDC0_{LN}^{Ag}M_{on}}$	Initial number of mDCs with medium antigen expression in LN (binding-phase)	0	#cells	-
$\boldsymbol{mDC0_{LN}^{Ag}H}$	Initial number of mDCs with high antigen expression in LN	0	#cells	-
$\boldsymbol{mDC0_{LN}^{Ag}M_{off}}$	Initial number of mDCs with medium antigen expression in LN (unbinding-phase)	0	#cells	-
$\boldsymbol{mDC0_{LN}^{Ag}L_{off}}$	Initial number of mDCs with low antigen expression in LN (unbinding-phase)	0	#cells	-
$\boldsymbol{mDC0_{LN}^{off}}$	Initial number of mature mDCs with no more antigen expression in LN	0	#cells	-
$\boldsymbol{pDC0_{LN}^{LNP}}$	Initial number of pDCs that internalized the LNPs in LN	0	#cells	-
$\boldsymbol{pDC0_{LN}^{Ag}L_{on}}$	Initial number of pDCs with low antigen expression in LN (binding-phase)	0	#cells	-
$\boldsymbol{pDC0_{LN}^{Ag}M_{on}}$	Initial number of pDCs with medium antigen expression in LN (binding-phase)	0	#cells	-
$\boldsymbol{pDC0_{LN}^{Ag}H}$	Initial number of pDCs with high antigen expression in LN	0	#cells	-
$\boldsymbol{pDC0_{LN}^{Ag}M_{off}}$	Initial number of pDCs with medium antigen expression in LN (unbinding-phase)	0	#cells	-
$\boldsymbol{pDC0_{LN}^{Ag}L_{off}}$	Initial number of pDCs with low antigen expression in LN (unbinding-phase)	0	#cells	-
$\boldsymbol{pDC0_{LN}^{off}}$	Initial number of mature pDCs with no more antigen expression in LN	0	#cells	-
$\boldsymbol{NT0}$	Initial number of naïve T-cells in the injection site	1445	#cells	[3]
$\boldsymbol{AT_{N0}}$	Initial number of activated T-cells from naïve T-cells	0	#cells	-
$\boldsymbol{MT0}$	Initial number of memory T-cells	0	#cells	-
$\boldsymbol{AT_{M0}}$	Initial number of activated T-cells from memory T helper cells	0	#cells	-
$\boldsymbol{FT0}$	Initial number of functional T-cell	0	#cells	-
$\boldsymbol{NB0}$	Initial number of naïve B-cells in LN	from 8.6537 to 778.9853	#cells	[3]
$\boldsymbol{AB_{N0}}$	Initial number of activated B-cells from naïve B cells	0	#cells	-
$\boldsymbol{GCB0}$	Initial number of activated B-cells in germinal center	0	#cells	-
$\boldsymbol{BM0}$	Initial number of memory B-cells	0	#cells	-
$\boldsymbol{AB_{M0}}$	Initial number of activated B-cells from memory B cells	)0	#cells	-
$\boldsymbol{SP0}$	Initial number of short-lived plasma cells in LN	0	#cells	-

$LP0$	Initial number of long-lived plasma cells in LN	0	#cells	-
**Blood (BL)**				
$NP0_{BL}$	Initial number of naïve NPs in BL	1.5950e+10	#cells	[4]
$MN0_{BL}$	Initial number of naïve MNs in BL	1.8800e+09	#cells	[5]
$mDC0_{BL}$	Initial number of naïve mDCs in BL	6.1925e+07	#cells	[5]
$pDC0_{BL}$	Initial number of naïve pDCs in BL	3.5e+07	#cells	[5]
$SP0_{BL}$	Initial number of short-lived plasma cells in BL	0	#cells	-
$LP0_{BL}$	Initial number of long-lived plasma cells in BL	0	#cells	-
Ab_0	Initial number of antibodies in BL	0	pmol	-

Table S2: *initial values of the tissue layer variables.*

S1.3 MODEL PARAMETERS

Parameter	Description	Estimate	Units	Reference
**General parameters**				
k_{dmRNA}	mRNA degradation rate	1.6636	day^{-1}	[3]
$MHC0$	Number of MHC in a single mature DC	1.5792e-07	#molecules	[4]
V_{LN}	Lymph node volume	5.0000e-04	L	[5]
V_{BL}	Blood volume in human	5.5	L	[6]
N_A	Avogadro constant	6.0220e+23	#molecules /mole	[7]
k_{deg}	Internalization and degradation rates of LNPs by muscle uptake	0.1186	day^{-1}	Fitted on Liang data[2]
$mRNA_{max}$	Quantity of mRNA triggering a regulatory response at IS	3.1416	pmol	Fitted on antibodies data [8–10]
k_{sat}	Rate of mRNA degradation due to the regulatory mechanisms	55.8330	day^{-1}	Fitted on antibodies data [8–10]
k_{slope}	Slope of the regulatory function	1	pmol	Model assumption to adimensionalize the term
K_{mRNA}	mRNA concentration required to achieve 50% recruitment rate of naïve APCs	0.2072	pmol	Fitted on Liang data[2]
exp_{Ag}	Scaling factor for antigen quantity in LN	3.3713e+05	pure number	Fitted on antibodies data [8–10]
SF_{mRNA}	Scaling factor for mRNA uptake rate	2.9587e+03	pmol^{-1}	Fitted on Liang data[2]
**Dose parameters for BNT162b2 (Pfizer vaccine)**				
$mRNA0_g$	mRNA dose	3.0000e-05	g	[1]
Ag_{MW}	Single strand molecular weight of BNT162b2 mRNA sequence	1.3775e+06	g/mol	Evaluated with Cusabio molecular weight calculator[11]
**Neutrophil (NPs) parameters**				
k_{dt}^{NP}	NPs death rate	2.3765	day^{-1}	[4]

k_{rc}^{NP}	NPs recruitment rate (function of mRNA)	8.6040e-04	day^{-1}	Fitted on Liang data[2]
k_{up}^{NP}	NPs internalizing LNPs	2.3216e-06	day^{-1}	Fitted on Liang data[2]
k_{exp}^{NP}	NPs antigen expression rate	0.1822	day^{-1}	Fitted on Liang data[2]
$k_{IS2LN}^{NP^{LNP}}$	NPs that internalized the LNPs migration rate from IS to LN	0.1822	day^{-1}	Fitted on Liang data[2]
$k_{IS2LN}^{NP^{Ag}}$	Expressing antigen NPs migration rate from IS to LN	0.1112	day^{-1}	Fitted on Liang data[2]
k_{IS2BL}^{NP}	Naïve NPs migration rate from IS to BL	4.1028e-04	day^{-1}	Fitted on Liang data[2]
$k_{br}^{NP_{IS}}$	Naïve NPs generation rate at IS	0	day^{-1}	Computed to preserve steady state without vaccination
$k_{br}^{NP_{BL}}$	Naïve NPs generation rate in BL	3.7905e+10	day^{-1}	Computed to preserve steady state without vaccination
**Monocyte (MNs) parameters**				
k_{dt}^{MN}	MNs Death rate	0.6931	day^{-1}	[4]
k_{rc}^{MN}	MNs recruitment rate (function of mRNA)	0.0038	day^{-1}	Fitted on Liang data[2]
k_{up}^{MN}	MNs internalizing LNPs	4.0593e-06	day^{-1}	Fitted on Liang data[2]
k_{exp}^{MN}	MNs antigen expression rate	4.3040	day^{-1}	Fitted on Liang data[2]
$k_{IS2LN}^{MN^{LNP}}$	MNs that internalized the LNPs migration rate from IS to LN	0.5219	day^{-1}	Fitted on Liang data[2]
$k_{IS2LN}^{MN^{Ag}}$	Expressing antigen NPs migration rate from IS to LN	1.1946e-04	day^{-1}	Fitted on Liang data[2]
k_{IS2BL}^{MN}	Naïve MNs migration rate from IS to BL	0.0060	day^{-1}	Fitted on Liang data[2]
$k_{br}^{MN_{IS}}$	Naïve MNs generation rate at IS	208.1375	day^{-1}	Computed to preserve steady state without vaccination
$k_{br}^{MN_{BL}}$	Naïve MNs generation rate in BL	1.3031e+09	day^{-1}	Computed to preserve steady state without vaccination
**Dendritic Cell (DCs) parameters**				
p_L	Low antigen expression maturation weight	0.2	pure number	Model assumption
p_M	Medium antigen expression maturation weight	0.65	pure number	Model assumption
p_H	High antigen expression maturation weight	1	pure number	Model assumption
**Myeloid Dendritic Cell (mDCs) parameters**				
k_{exp}^{mDC}	mDCs maturation rate from mDC^{LNP} to $mDC^{Ag}L_{on}$	4.9219	day^{-1}	Fitted on Liang data[2]

$k_{tr}^{mDC,M}$	mDCs maturation rate from $mDC^{Ag}L_{on}$ to $mDC^{Ag}M_{on}$	5.9867	day^{-1}	Derived as reported in Supplementary Section S3
$k_{tr}^{mDC,H}$	mDCs maturation rate from $mDC^{Ag}M_{on}$ to $mDC^{Ag}H$	4.0242	day^{-1}	Derived as reported in Supplementary Section S3
$k_{atr}^{mDC,H}$	mDCs maturation rate from $mDC^{Ag}H$ to $mDC^{Ag}M_{off}$	2.0006	day^{-1}	Derived as reported in Supplementary Section S3
$k_{atr}^{mDC,M}$	mDCs maturation rate from $mDC^{Ag}M_{off}$ to $mDC^{Ag}L_{off}$	1.1825	day^{-1}	Derived as reported in Supplementary Section S3
$k_{atr}^{mDC,L}$	mDCs maturation rate from $mDC^{Ag}L_{off}$ to mDC^{off}	0.3939	day^{-1}	Derived as reported in Supplementary Section S3
k_{dt}^{ImDC}	Naïve mDCs death rate	0.0924	day^{-1}	[4]
k_{dt}^{mDC}	Mature mDCs death rate	1.1402	day^{-1}	Fitted on Liang data[2]
k_{rc}^{mDC}	mDCs recruitment rate (function of $mRNA_{IS}$)	0.0052	day^{-1}	Fitted on Liang data[2]
k_{up}^{mDC}	mDCs internalizating LNPs	5.0855e-06	day^{-1}	Fitted on Liang data[2]
$k_{IS2LN}^{mDC^{LNP}}$	mDCs migration rate from IS to LN of mDC^{LNP}	0.1468	day^{-1}	Fitted on Liang data[2]
$k_{IS2LN}^{mDC^{Ag}}$	mDCs migration rate from IS to LN with antigen presentation	0.2351	day^{-1}	Fitted on Liang data[2]
k_{IS2BL}^{mDC}	Naïve mDCs migration rate from IS to BL	0.2449	day^{-1}	Fitted on Liang data[2]
$k_{br}^{mDC_{IS}}$	Naïve mDCs generation rate at IS	1.0827e+03	day^{-1}	Computed to preserve steady state without vaccination
$k_{br}^{mDC_{BL}}$	Naïve mDCs generation rate in BL	5.7210e+06	day^{-1}	Computed to preserve steady state without vaccination
**Plasmacytoid Dendritic Cells (pDC) parameters**				
k_{exp}^{pDC}	pDCs maturation rate from pDC^{LNP} to $pDC^{Ag}L_{on}$	29.1598	day^{-1}	Fitted on Liang data[2]
$k_{tr}^{pDC,M}$	pDCs maturation rate from $pDC^{Ag}L_{on}$ to $pDC^{Ag}M_{on}$	5.9887	day^{-1}	Derived as reported in Supplementary Section S3
$k_{tr}^{pDC,H}$	mDCs maturation rate from $mDC^{Ag}M_{on}$ to $mDC^{Ag}H$	4.0250	day^{-1}	Derived as reported in Supplementary Section S3
$k_{atr}^{pDC,H}$	pDCs maturation rate from $pDC^{Ag}H$ to $pDC^{Ag}M_{off}$	2.0004	day^{-1}	Derived as reported in Supplementary Section S3
$k_{atr}^{pDC,M}$	pDCs maturation rate from $pDC^{Ag}M_{off}$ to $pDC^{Ag}L_{off}$	1.1824	day^{-1}	Derived as reported in Supplementary Section S3

$k_{atr}^{pDC,L}$	pDCs maturation rate from $pDC^{Ag}L_{off}$ to pDC^{off}	0.3939	day^{-1}	Derived as reported in Supplementary Section S3
k_{dt}^{IpDC}	Naïve pDCs Death rate	0.0924	day^{-1}	[4]
k_{dt}^{pDC}	mature pDCs death rate	1.3501	day^{-1}	Fitted on Liang data[2]
k_{rc}^{pDC}	pDCs recruitment rate (function of $mRNA_{IS}$)	4.9994e-04	day^{-1}	Fitted on Liang data[2]
k_{up}^{pDC}	pDCs inbternalizing LNPs	4.4309e-06	day^{-1}	Fitted on Liang data[2]
$k_{IS2LN}^{pDC^{LNP}}$	pDCs migration rate from IS to LN of pDC^{LNP}	27.0726	day^{-1}	Fitted on Liang data[2]
$k_{IS2LN}^{pDC^{Ag}}$	pDCs migration rate from IS to LN with antigen presentation	0.0039	day^{-1}	Fitted on Liang data[2]
k_{IS2BL}^{pDC}	Naïve pDCs migration rate from IS site to BL	0.1548	day^{-1}	Fitted on Liang data[2]
$k_{br}^{pDC_{IS}}$	Naïve pDCs generation rate at IS	0	day^{-1}	Computed to preserve steady state without vaccination
$k_{br}^{pDC_{BL}}$	Naïve pDCs generation rate in BL	3.2340e+06	day^{-1}	Computed to preserve steady state without vaccination
**T-cell parameters**				
K_{NT}	number of T-epitope-MHC II-peptide complexes on DCs membrane required to achieve 50% activation rate of naïve T-cell activation	400	#epitopes	[4]
K_{MT}	number of T-epitope-MHC II-peptide complexes on DCs membrane required to achieve 50% activation rate of memory T-cell activation	40	#epitopes	[4]
T_{act}	Minimum number of DCs required for 1 T-cell activation (more than 50% chances of activation)	100	#cells	[12]
k_{dt}^{NT}	Death rate of naïve T-cells	0.0029	day^{-1}	[4]
k_{dt}^{AT}	Death rate of activated T-cells	0.18	day^{-1}	[4]
k_{dt}^{MT}	Death rate of memory T-cells	2.7397e-04	day^{-1}	[4]
k_{dt}^{FT}	Death rate of functional T-cells	0.1800	day^{-1}	[4]
k_{act}^{NT}	Maximum activation rate for naïve T-cells	294.6588	day^{-1}	Fitted on antibodies data[8–10]
k_{act}^{MT}	Maximum activation rate for memory T-cells	935.8438	day^{-1}	Fitted on antibodies data[8–10]
k_{prol}^{AT}	Maximum proliferation rate for activated T-cells	4.9809	day^{-1}	Fitted on antibodies data[8–10]

f^{AT}	Percentage of activated T-cells differentiating into memory T-cells	0.5	pure number	Model assumption
**B-cell parameters**				
BRN	Number of receptors on each B-cell	75000	#molecules /cell	[4]
J	Number of B-cell subclones (with different affinities)	17	#cells	[4]
$K_{a,i}$	Association rate constant for Ag-BCR/Ag-Ab binding	3.9063e-09 - 2.56e-04	pM^{-1}	[4]
K_R	Occupied BCRs to achieve 50% activation rate of naïve B-cells	1	pure number	[4]
CC_N	Carrying capacity of 1 functional T cell to stimulate activation and proliferation of naïve B cells	66.1786	pure number	Fitted on antibodies data[8–10]
CC_M	Carrying capacity of 1 functional T cell to stimulate activation and proliferation of memory B-cells	661.786 (= 10 times the value of CC_N)	pure number	Model assumption
$k_{prol}^{AB_N}$	Maximum proliferation rate of activated B-cells differentiated from naïve B-cells	6.0612	day^{-1}	Fitted on antibodies data[8–10]
$k_{prol}^{AB_M}$	Maximum proliferation rate of activated B-cells differentiated from memory B-cells	6.3443	day^{-1}	Fitted on antibodies data[8–10]
k_{dt}^{AB}	Death rate of activated B-cells	0.2518	day^{-1}	[4]
k_{dt}^{MB}	Death rate of naïve B-cells	0.029	day^{-1}	[13–15]
k_{dt}^{MB}	Death rate of memory B-cells	7.8278e-05	day^{-1}	[4]
k_{dt}^{SP}	Death rate of short-lived plasma cells	0.1383	day^{-1}	Fitted on antibodies data[8–10]
k_{dt}^{LP}	Death rate of long-lived plasma cells	0.0143	day^{-1}	Fitted on antibodies data[8–10]
g_1	Percentage of activated B-cells differentiating into memory B-cells	0.5	pure number	[4]
g_2	Percentage for activated B-cells differentiating into short-lived plasma cells	0.4	pure number	[4]
k_{act}^{NB}	Maximum activation rate of naïve B-cells	2.48	day^{-1}	[13]
k_{act}^{MB}	Maximum activation rate of memory B-cells	8.6212	day^{-1}	Fitted on antibodies data[8–10]
$delay_B$	Activated B-cell migration rate to germinal center	7.9506e-07	day^{-1}	Fitted on antibodies data[8–10]
k_{LN2BL}^{PC}	Plasma cell migration rate from LN to BL	40.4821	day^{-1}	Fitted on antibodies data[8–10]
**Antibodies (Ab) parameters**				
k_{prod}^{Ab}	Plasma cell secretion rate of Ab in BL	8.64+e08	day^{-1}	[4]

$\boldsymbol{k_{deg}^{Ab}}$	Degradation rate of Ab in BL	0.0918	day^{-1}	Fitted on antibodies data[8–10]

Table S3: *parameters (fitted or retrieved from literature) of the tissue layer.*

S1.4 MODEL EQUATIONS

We present the complete set of ordinary differential equations (ODEs) employed to model the immune response following intramuscular injection of an mRNA vaccine. The model encompasses three different compartments: the injection site (IS), the draining lymph node (LN) and the blood (BL).

The antigen presenting cell (APC) populations considered in the model include neutrophils (NPs), monocytes (MNs), as well as two subsets of DCs: myeloid dendritic cells (mDCs) and plasmacytoid dendritic cells (pDCs).

The birth rates k_{br}^{cells} represent the physiological recruitment at steady state under the homeostatic assumption, defined as $k_{br}^{cells} = \text{cells0} \cdot \left(k_{dt}^{Icells} + k_{IS2BL}^{cells}\right)$, where $cells$ refers to any cell population, $cells0$ is the initial amount of naïve cells, k_{dt}^{Icells} is the death rate of immature (naïve) cells in the population, and k_{IS2BL}^{cells} is the migration rate from the injection site to the blood.

The adaptive immune response is modeled following established literature[4,16,17].

S1.4.1 Injection Site – IS

mRNA equation at the injection site

The following equation describes mRNA degradation and uptake by APCs at the injection site. The cells responsible for this uptake are NPs, MNs (non-classical, intermediate and classical monocytes), mDCs and pDCs. The rate k_{dmRNA} is derived from the mRNA half-life of approximately 10 hours. The term k_{deg} represents mRNA consumption by muscle cells. Additionally, the final term accounts for mRNA consumption by various actors when a high dose of the products is injected.

$$\begin{aligned}\frac{dmRNA_{IS}}{dt} = &- k_{up}^{NP} \cdot mRNA_{IS} \cdot NP_{IS} - k_{up}^{MN} \cdot mRNA_{IS} \cdot MN_{IS} - k_{up}^{mDC} \cdot mRNA_{IS} \cdot mDC_{IS} \\ &- k_{up}^{pDC} \cdot mRNA_{IS} \cdot pDC_{IS} - k_{dmRNA} \cdot mRNA_{IS} - k_{deg} \cdot mRNA_{IS} \\ &- k_{sat} \cdot mRNA_{IS} \cdot \frac{1}{2}\,(1 + \tanh \frac{(mRNA_{IS} - mRNA_{max})}{k_{slope}})\end{aligned}$$

(1)

Neutrophils equation at the injection site

The following equation describes the recruitment of naïve NPs at the injection site (as a function of mRNA), their mRNA uptake, and the steady-state dynamics governed by generation, death and migration rates.

$$\begin{aligned}\frac{dNP_{IS}}{dt} = &\, k_{br}^{NP_{IS}} + k_{rc}^{NP} \cdot \frac{mRNA_{IS}}{K_{mRNA} + mRNA_{IS}} \cdot NP_{BL} - k_{up}^{NP} \cdot SF_{mRNA} \cdot mRNA_{IS} \cdot NP_{IS} - k_{dt}^{NP} \cdot NP_{IS} \\ &- k_{IS2BL}^{NP} \cdot NP_{IS}\end{aligned}$$

(2)

We present the equations describing the internalization of lipid nanoparticles (LNPs) and the subsequent translation of the antigen protein by neutrophils at the injection site:

$$\frac{dNP_{IS}^{LNP}}{dt} = k_{up} \cdot SF_{mRNA} \cdot m\text{RNA}_{IS} \cdot NP_{IS} - k_{exp}^{NP} \cdot NP_{IS}^{LNP} - k_{dt}^{NP} \cdot NP_{IS}^{LNP} - k_{IS2LN}^{NP^{LNP}} \cdot NP_{IS}^{LNP} \tag{3}$$

$$\frac{dNP_{IS}^{Ag}}{dt} = k_{exp}^{NP} \cdot NP_{IS}^{LNP} - k_{dt}^{NP} \cdot NP_{IS}^{Ag} - k_{IS2LN}^{NP^{Ag}} \cdot NP_{IS}^{Ag} \tag{4}$$

Monocytes equation at the injection site

The following equation describes the recruitment of naïve MNs at the injection site (as a function of mRNA), their mRNA uptake, and the steady-state dynamics governed by generation, death and migration rates.

$$\frac{dMN_{IS}}{dt} = k_{br}^{MN_{IS}} + k_{rc}^{MN} \cdot \frac{mRNA_{IS}}{K_{mRNA} + mRNA_{IS}} \cdot MN_{BL} - k_{up}^{MN} \cdot SF_{mRNA} \cdot m\text{RNA}_{IS} \cdot MN_{IS} - k_{dt}^{MN} \cdot MN_{IS} - k_{IS2BL}^{MN} \cdot MN_{IS} \tag{5}$$

We present the equations describing the internalization of LNPs and the subsequent translation of the antigen protein by MNs at the injection site:

$$\frac{dMN_{IS}^{LNP}}{dt} = k_{up} \cdot SF_{mRNA} \cdot m\text{RNA}_{IS} \cdot MN_{IS} - k_{exp}^{MN} \cdot MN_{IS}^{LNP} - k_{dt}^{MN} \cdot MN_{IS}^{LNP} - k_{IS2LN}^{MN^{LNP}} \cdot MN_{IS}^{LNP} \tag{6}$$

$$\frac{dMN_{IS}^{Ag}}{dt} = k_{exp}^{MN} \cdot MN_{IS}^{LNP} - k_{dt}^{MN} \cdot MN_{IS}^{Ag} - k_{IS2LN}^{MN^{Ag}} \cdot MN_{IS}^{Ag} \tag{7}$$

Myeloid dendritic cells equation at the injection site

The following equation describes the recruitment of naïve mDCs at the injection site (as a function of mRNA), their mRNA uptake, and the steady-state dynamics governed by generation, death and migration rates:

$$\frac{dmDC_{IS}}{dt} = k_{br}^{mDC_{IS}} + k_{rc}^{mDC} \cdot \frac{mRNA_{IS}}{K_{mRNA} + mRNA_{IS}} \cdot m\text{DC}_{BL} - k_{up}^{mDC} \cdot SF_{mRNA} \cdot m\text{RNA}_{IS} \cdot mDC_{IS} - k_{dt}^{ImDC} \cdot m\text{DC}_{IS} - k_{IS2BL}^{mDC} \cdot m\text{DC}_{IS} \tag{8}$$

We model the internalization of LNPs and the maturation process of DCs. Upon LNP uptake the mDCs initiate the translation and surface presentation of the antigen protein on their plasma membrane. The level of antigen presentation increases over time, progressing from low to medium and high levels, followed by a decline back to medium and low due to the unbinding and degradation of MHC II-Ag complexes. The maturation rates are optimized based on the molecular layer model (see **Section S3**).

$$\frac{dmDC_{IS}^{LNP}}{dt} = k_{up} \cdot SF_{mRNA} \cdot m\text{RNA}_{IS} \cdot mDC_{IS} - k_{exp}^{mDC} \cdot mDC_{IS}^{LNP} - k_{dt}^{mDC} \cdot mDC_{IS}^{LNP} - k_{IS2LN}^{mDC^{LNP}} \cdot mDC_{IS}^{LNP} \tag{9}$$

$$\frac{dmDC_{IS}^{Ag}L_{on}}{dt} = k_{exp}^{mDC} \cdot \mathrm{mDC}_{IS}^{LNP} - k_{tr}^{M} \cdot \mathrm{mDC}_{IS}^{Ag}L_{on} - k_{dt}^{mDC} \cdot \mathrm{mDC}_{IS}^{Ag}L_{on} - k_{IS2LN}^{mDC^{Ag}} \cdot \mathrm{mDC}_{IS}^{Ag}L_{on}$$

(10)

$$\frac{dmDC_{IS}^{Ag}M_{on}}{dt} = k_{tr}^{M} \cdot \mathrm{mDC}_{IS}^{Ag}L_{on} - k_{tr}^{H} \cdot mDC_{IS}^{Ag}M_{on} - k_{dt}^{mDC} \cdot \mathrm{mDC}_{IS}^{Ag}M_{on} - k_{IS2LN}^{mDC^{Ag}} \cdot \mathrm{mDC}_{IS}^{Ag}M_{on}$$

(11)

$$\frac{dmDC_{IS}^{Ag}H}{dt} = k_{tr}^{H} \cdot \mathrm{mDC}_{IS}^{Ag}M_{on} - k_{atr}^{H} \cdot \mathrm{mDC}_{IS}^{Ag}H - k_{dt}^{mDC} \cdot \mathrm{mDC}_{IS}^{Ag}H - k_{IS2LN}^{mDC^{Ag}} \cdot \mathrm{mDC}_{IS}^{Ag}H$$

(12)

$$\frac{dmDC_{IS}^{Ag}M_{off}}{dt} = k_{atr}^{H} \cdot \mathrm{mDC}_{IS}^{Ag}H - k_{atr}^{M} \cdot \mathrm{mDC}_{IS}^{Ag}M_{off} - k_{dt}^{mDC} \cdot \mathrm{mDC}_{IS}^{Ag}M_{off} - k_{IS2LN}^{mDC^{Ag}} \cdot \mathrm{mDC}_{IS}^{Ag}M_{off}$$

(13)

$$\frac{dmDC_{IS}^{Ag}L_{off}}{dt} = k_{atr}^{M} \cdot \mathrm{mDC}_{IS}^{Ag}\mathrm{M_{off}} - k_{atr}^{L} \cdot \mathrm{mDC}_{IS}^{Ag}L_{off} - k_{dt}^{mDC} \cdot \mathrm{mDC}_{IS}^{Ag}L_{off} - k_{IS2LN}^{mDC^{Ag}} \cdot \mathrm{mDC}_{IS}^{Ag}L_{off}$$

(14)

$$\frac{dmDC_{IS}^{off}}{dt} = k_{atr}^{L} \cdot \mathrm{mDC}_{IS}^{Ag}\mathrm{L_{off}} - k_{dt}^{mDC} \cdot \mathrm{mDC}_{IS}^{off} - k_{IS2LN}^{mDC^{Ag}} \cdot \mathrm{mDC}_{IS}^{off}$$

(15)

Plasmacytoid dendritic cells equation at the injection site

The following equation describes the recruitment of naïve pDCs at the injection site (as a function of mRNA), their mRNA uptake, and the steady-state dynamics governed by generation, death and migration rates:

$$\frac{dpDC_{IS}}{dt} = k_{br}^{pDC_{IS}} + k_{rc}^{pDC} \cdot \frac{mRNA_{IS}}{K_{mRNA} + mRNA_{IS}} \cdot \mathrm{pDC}_{BL} - k_{up}^{pDC} \cdot SF_{mRNA} \cdot mRNA_{IS} \cdot pDC_{IS} - k_{dt}^{IpDC} \cdot pDC_{IS} - k_{IS2BL}^{pDC} \cdot pDC_{IS}$$

(16)

The following equations describe the internalization of LNPs and the maturation process of DCs. Upon LNP uptake, the pDCs initiate the translation and surface exposure of the antigen protein on their plasma membrane. The level of antigen presentation gradually increases from low to medium and high, followed by a decline back to medium and low due to the unbinding and degradation of MHC II-Ag complexes. The maturation rates are optimized based on the molecular layer model (see **Section S3**).

$$\frac{dpDC_{IS}^{LNP}}{dt} = k_{up} \cdot SF_{mRNA} \cdot mRNA_{IS} \cdot \mathrm{pDC}_{IS} - k_{exp}^{pDC} \cdot \mathrm{pDC}_{IS}^{LNP} - k_{dt}^{pDC} \cdot \mathrm{pDC}_{IS}^{LNP} - k_{IS2LN}^{pDC^{LNP}} \cdot \mathrm{pDC}_{IS}^{LNP}$$

(17)

$$\frac{dpDC_{IS}^{Ag}L_{on}}{dt} = k_{exp}^{pDC} \cdot \mathrm{pDC}_{IS}^{LNP} - k_{tr}^{M} \cdot \mathrm{pDC}_{IS}^{Ag}L_{on} - k_{dt}^{pDC} \cdot \mathrm{pDC}_{IS}^{Ag}L_{on} - k_{IS2LN}^{pDC^{Ag}} \cdot \mathrm{pDC}_{IS}^{Ag}L_{on}$$

(18)

$$\frac{dpDC_{IS}^{Ag}M_{on}}{dt} = k_{tr}^{M} \cdot pDC_{IS}^{Ag}L_{on} - k_{tr}^{H} \cdot pDC_{IS}^{Ag}M_{on} - k_{dt}^{pDC} \cdot pDC_{IS}^{Ag}M_{on} - k_{IS2LN}^{pDC^{Ag}} \cdot pDC_{IS}^{Ag}M_{on} \tag{19}$$

$$\frac{dpDC_{IS}^{Ag}H}{dt} = k_{tr}^{H} \cdot pDC_{IS}^{Ag}M_{on} - k_{atr}^{H} \cdot pDC_{IS}^{Ag}\mathrm{H} - k_{dt}^{pDC} \cdot pDC_{IS}^{Ag}\mathrm{H} - k_{IS2LN}^{pDC^{Ag}} \cdot pDC_{IS}^{Ag}\mathrm{H} \tag{20}$$

$$\frac{dpDC_{IS}^{Ag}M_{off}}{dt} = k_{atr}^{H} \cdot pDC_{IS}^{Ag}\mathrm{H} - k_{atr}^{M} \cdot pDC_{IS}^{Ag}M_{off} - k_{dt}^{pDC} \cdot pDC_{IS}^{Ag}M_{off} - k_{IS2LN}^{pDC^{Ag}} \cdot pDC_{IS}^{Ag}M_{off} \tag{21}$$

$$\frac{dpDC_{IS}^{Ag}L_{off}}{dt} = k_{atr}^{M} \cdot pDC_{IS}^{Ag}\mathrm{M_{off}} - k_{atr}^{L} \cdot pDC_{IS}^{Ag}L_{off} - k_{dt}^{pDC} \cdot pDC_{IS}^{Ag}L_{off} - k_{IS2LN}^{pDC^{Ag}} \cdot pDC_{IS}^{Ag}L_{off} \tag{22}$$

$$\frac{dpDC_{IS}^{off}}{dt} = k_{atr}^{L} \cdot pDC_{IS}^{Ag}\mathrm{L_{off}} - k_{dt}^{pDC} \cdot pDC_{IS}^{off} - k_{IS2LN}^{pDC^{Ag}} \cdot pDC_{IS}^{off} \tag{23}$$

S1.4.2 Draining Lymph Node - LN

Neutrophils equation at draining lymph node

Equations describing the NPs internalizing LNPs and those translating the antigen as they migrate from the injection site to the draining lymph node:

$$\frac{dNP_{LN}^{LNP}}{dt} = k_{IS2LN}^{NP^{LNP}} \cdot NP_{IS}^{LNP} - k_{exp}^{NP} \cdot NP_{LN}^{LNP} - k_{dt}^{NP} \cdot NP_{LN}^{LNP} \tag{24}$$

$$\frac{dNP_{LN}^{Ag}}{dt} = k_{IS2LN}^{NP^{Ag}} \cdot NP_{IS}^{Ag} + k_{exp}^{NP} \cdot NP_{LN}^{LNP} - k_{dt}^{NP} \cdot NP_{LN}^{Ag} \tag{25}$$

Monocytes equation at the draining lymph node

Equations describing the MNs internalizing LNPs and those translating the antigen as they migrate from the injection site to the draining lymph node:

$$\frac{dMN_{LN}^{LNP}}{dt} = k_{IS2LN}^{MN^{LNP}} \cdot MN_{IS}^{LNP} - k_{exp}^{MN} \cdot MN_{LN}^{LNP} - k_{dt}^{MN} \cdot MN_{LN}^{LNP} \tag{26}$$

$$\frac{dMN_{LN}^{Ag}}{dt} = k_{IS2LN}^{MN^{Ag}} \cdot MN_{IS}^{Ag} + k_{exp}^{MN} \cdot MN_{LN}^{LNP} - k_{dt}^{MN} \cdot MN_{LN}^{Ag} \tag{27}$$

Myeloid dendritic cells equation at the draining lymph node

We model the migration of mDCs from the injection site to the draining lymph node after internalizing LNPs and undergoing the maturation process. Following LNP uptake, mDCs initiate the translation and surface presentation of the antigen protein on their plasma membrane. The level of antigen presentation increases

from low to medium and high, followed by a decline back to medium and low due to the unbinding and degradation of MHC II-Ag complexes. The maturation rates are optimized based on the molecular layer model (see **Section S3**).

$$\frac{dmDC_{LN}^{LNP}}{dt} = k_{IS2LN}^{mDC^{LNP}} \cdot mDC_{IS}^{LNP} - k_{exp}^{mDC} \cdot \mathrm{mDC}_{LN}^{LNP} - k_{dt}^{mDC} \cdot \mathrm{mDC}_{LN}^{LNP} \tag{28}$$

$$\frac{dmDC_{LN}^{Ag}L_{on}}{dt} = k_{IS2LN}^{mDC^{Ag}} \cdot mDC_{IS}^{Ag}L_{on} + k_{exp}^{mDC} \cdot \mathrm{mDC}_{LN}^{LNP} - k_{tr}^{M} \cdot \mathrm{mDC}_{LN}^{Ag}L_{on} - k_{dt}^{mDC} \cdot \mathrm{mDC}_{LN}^{Ag}L_{on} \tag{29}$$

$$\frac{dmDC_{LN}^{Ag}M_{on}}{dt} = k_{IS2LN}^{mDC^{Ag}} \cdot mDC_{IS}^{Ag}M_{on} + k_{tr}^{M} \cdot \mathrm{mDC}_{LN}^{Ag}L_{on} - k_{tr}^{H} \cdot \mathrm{mDC}_{LN}^{Ag}M_{on} - k_{dt}^{mDC} \cdot \mathrm{mDC}_{LN}^{Ag}M_{on} \tag{30}$$

$$\frac{dmDC_{LN}^{Ag}H}{dt} = k_{IS2LN}^{mDC^{Ag}} \cdot mDC_{IS}^{Ag}H + k_{tr}^{H} \cdot \mathrm{mDC}_{LN}^{Ag}M_{on} - k_{atr}^{H} \cdot \mathrm{mDC}_{LN}^{Ag}\mathrm{H} - k_{dt}^{mDC} \cdot \mathrm{mDC}_{LN}^{Ag}\mathrm{H} \tag{31}$$

$$\frac{dmDC_{LN}^{Ag}M_{off}}{dt} = k_{IS2LN}^{mDC^{Ag}} \cdot mDC_{IS}^{Ag}M_{off} + k_{atr}^{H} \cdot \mathrm{mDC}_{LN}^{Ag}\mathrm{H} - k_{atr}^{M} \cdot \mathrm{mDC}_{LN}^{Ag}M_{off} - k_{dt}^{mDC} \cdot \mathrm{mDC}_{LN}^{Ag}M_{off} \tag{32}$$

$$\frac{dmDC_{LN}^{Ag}L_{off}}{dt} = k_{IS2LN}^{mDC^{Ag}} \cdot mDC_{IS}^{Ag}L_{off} + k_{atr}^{M} \cdot \mathrm{mDC}_{LN}^{Ag}\mathrm{M_{off}} - k_{atr}^{L} \cdot \mathrm{mDC}_{LN}^{Ag}L_{off} - k_{dt}^{mDC} \cdot \mathrm{mDC}_{LN}^{Ag}L_{off} \tag{33}$$

$$\frac{dmDC_{LN}^{off}}{dt} = k_{IS2LN}^{mDC^{Ag}} \cdot mDC_{IS}^{off} + k_{atr}^{L} \cdot \mathrm{mDC}_{LN}^{Ag}\mathrm{L_{off}} - k_{dt}^{mDC} \cdot mDC_{LN}^{off} \tag{34}$$

Plasmacytoid dendritic cells equation at the draining lymph node

We model the migration of pDCs from the injection site to the draining lymph node after internalizing LNPs and undergoing the maturation process. Following LNP uptake, pDCs initiate the translation and surface presentation of the antigen protein on their plasma membrane. The level of antigen presentation increases from low to medium and high, followed by a decline back to medium and low due to the unbinding and degradation of MHC II-Ag complexes. The maturation rates are optimized based on the molecular layer model (see **Section S3**).

$$\frac{dpDC_{LN}^{LNP}}{dt} = k_{IS2LN}^{pDC^{LNP}} \cdot pDC_{IS}^{LNP} - k_{exp}^{pDC} \cdot \mathrm{pDC}_{LN}^{LNP} - k_{dt}^{pDC} \cdot \mathrm{pDC}_{LN}^{LNP} \tag{35}$$

$$\frac{dpDC_{LN}^{Ag}L_{on}}{dt} = k_{IS2LN}^{pDC^{Ag}} \cdot pDC_{IS}^{Ag}L_{on} + k_{exp}^{pDC} \cdot \mathrm{pDC}_{LN}^{LNP} - k_{tr}^{M} \cdot \mathrm{pDC}_{LN}^{Ag}L_{on} - k_{dt}^{pDC} \cdot \mathrm{pDC}_{LN}^{Ag}L_{on} \tag{36}$$

$$\frac{dpDC_{LN}^{Ag}M_{on}}{dt} = k_{IS2LN}^{pDC^{Ag}} \cdot pDC_{IS}^{Ag}M_{on} + k_{tr}^{M} \cdot \mathrm{pDC}_{LN}^{Ag}L_{on} - k_{tr}^{H} \cdot \mathrm{pDC}_{LN}^{Ag}M_{on} - k_{dt}^{pDC} \cdot \mathrm{pDC}_{LN}^{Ag}M_{on}$$

(37)

$$\frac{dpDC_{LN}^{Ag}H}{dt} = k_{IS2LN}^{pDC^{Ag}} \cdot pDC_{LN}^{Ag}Hk_{tr}^{H} \cdot \mathrm{pDC}_{LN}^{Ag}M_{on} - k_{atr}^{H} \cdot \mathrm{pDC}_{LN}^{Ag}\mathrm{H} - k_{dt}^{pDC} \cdot \mathrm{pDC}_{LN}^{Ag}\mathrm{H}$$

(38)

$$\frac{dpDC_{LN}^{Ag}M_{off}}{dt} = k_{IS2LN}^{pDC^{Ag}} \cdot pDC_{IS}^{Ag}M_{off} + k_{atr}^{H} \cdot \mathrm{pDC}_{LN}^{Ag}\mathrm{H} - k_{atr}^{M} \cdot \mathrm{pDC}_{LN}^{Ag}M_{off} - k_{dt}^{pDC} \cdot \mathrm{pDC}_{LN}^{Ag}M_{off}$$

(39)

$$\frac{dpDC_{LN}^{Ag}L_{off}}{dt} = k_{IS2LN}^{pDC^{Ag}} \cdot pDC_{IS}^{Ag}L_{off} + k_{atr}^{M} \cdot \mathrm{pDC}_{LN}^{Ag}\mathrm{M_{off}} - k_{atr}^{L} \cdot \mathrm{pDC}_{LN}^{Ag}L_{off} - k_{dt}^{pDC} \cdot \mathrm{pDC}_{LN}^{Ag}L_{off}$$

(40)

$$\frac{dpDC_{LN}^{off}}{dt} = k_{IS2LN}^{pDC^{Ag}} \cdot pDC_{IS}^{off} + k_{atr}^{L} \cdot \mathrm{pDC}_{LN}^{Ag}\mathrm{L_{off}} - k_{dt}^{pDC} \cdot \mathrm{pDC}_{LN}^{off}$$

(41)

<u>Dendritic cells count at the draining lymph node</u>

To model the contribution of DCs to the activation of the adaptive immune system, we estimate the amount of antigen exposed on their plasma membrane.

Initially, we compute the total number of DCs present in the lymph node:

$$\mathrm{mD}C_{tot} = mDC_{LN}^{Ag}L_{on} + \mathrm{mDC}_{LN}^{Ag}L_{off} + \mathrm{mDC}_{LN}^{Ag}M_{on} + \mathrm{mDC}_{LN}^{Ag}M_{off} + \mathrm{mDC}_{LN}^{Ag}\mathrm{H}$$

(42)

$$\mathrm{pD}C_{tot} = \mathrm{pDC}_{LN}^{Ag}L_{on} + \mathrm{pDC}_{LN}^{Ag}L_{off} + \mathrm{pDC}_{LN}^{Ag}M_{on} + \mathrm{pDC}_{LN}^{Ag}M_{off} + \mathrm{pDC}_{LN}^{Ag}\mathrm{H}$$

(43)

Subsequently, we estimate the quantity of mature DCs within the lymph node, assigning distinct weights to reflect the different maturation levels of these cells:

$$\mathrm{mD}C_{mat} = \left(mDC_{LN}^{Ag}L_{on} + mDC_{LN}^{Ag}L_{off}\right) \cdot p_L + \left(mDC_{LN}^{Ag}M_{on} + mDC_{LN}^{Ag}M_{off}\right) \cdot p_M + \mathrm{mDC}_{LN}^{Ag}\mathrm{H} \cdot p_H$$

(44)

$$\mathrm{pD}C_{mat} = \left(pDC_{LN}^{Ag}L_{on} + pDC_{LN}^{Ag}L_{off}\right) \cdot p_L + \left(pDC_{LN}^{Ag}M_{on} + pDC_{LN}^{Ag}M_{off}\right) \cdot p_M + \mathrm{pDC}_{LN}^{Ag}\mathrm{H} \cdot p_H$$

(45)

We set mDC_{mat} and pDC_{mat} to 0 when the number of mature cells falls below the threshold for low antigen exposure, i.e. $\mathrm{mD}C_{mat} < p_L$ and $\mathrm{pD}C_{mat} < p_L$. Therefore, we define the cut-off as the situation in which at least one dendritic cell exhibits low antigen exposure, corresponding to 20% of MHC II molecules on the plasma membrane being occupied.

Finally, we calculate the average number of MHC II-Ag complexes presented on the DC plasma membrane. This is achieved by using the maximum number of MHC II-Ag complexes, denoted by $\max_{mDC}\mathrm{MHCIIAg}$ for myeloid dendritic cells and $\max_{pDC}\mathrm{MHCIIAg}$ for plasmatocytoid dendritic cells the molecular layer:

$$N_{\mathrm{mDC}} = \frac{mDC_{mat} \cdot \mathrm{max}_{\mathrm{mDC}} MHCIIAg}{mDC_{tot}}$$

(46)

$$N_{\mathrm{pDC}} = \frac{pDC_{mat} \cdot \mathrm{max}_{\mathrm{pDC}} MHCIIAg}{pDC_{tot}}$$

(47)

<u>T-cells equation at the draining lymph node - naïve T-cells activation equations</u>

To account for the contribution of APCs to the activation of naïve T-cells, we calculate a contact probability between a naïve T-cell and a maturing DC as $\frac{DC_{tot}}{DC_{tot}+NT+AT_N+AT_M+MT}$. subsequently, we compute the naïve T-cell activation rate as:

$$D_N^{mDC} = \frac{N_{mDC} \cdot \frac{mDC_{tot}}{mDC_{tot} + NT + AT_N + AT_M + MT}}{N_{mDC} + K_{NT}}$$

(48)

$$D_N^{pDC} = \frac{N_{pDC} \cdot \frac{pDC_{tot}}{pDC_{tot} + NT + AT_N + AT_M + MT}}{N_{pDC} + K_{NT}}$$

(49)

where K_{NT} represents the number of T-epitope-MHC II-peptide complexes on the DC membrane required to achieve a 50% activation rate of naïve helper T-cell.

Finally, we evaluate a weighted sum of the contributions from different DC populations to the activation of naïve T-cells as:

$$D_N = D_N^{mDC} \cdot w_{mDC} + D_N^{pDC} \cdot w_{pDC},$$

(50)

where the weights are computed as:

$$w_{mDC} = \frac{mDC_{tot}}{mDC_{tot} + pDC_{tot}}$$

(51)

$$w_{pDC} = \frac{pDC_{tot}}{mDC_{tot} + pDC_{tot}}$$

(52)

The weights w_{mDC} and w_{pDC} are set equal to 0 by default when the total number of mDCs and pDCs is zero, i.e., $mDC_{tot} + pDC_{tot} = 0$.

The equation modeling naïve T-cells activation is:

$$\frac{dNT}{dt} = k_{dt}^{NT} \cdot (NT0 - NT) - k_{act}^{NT} \cdot D_N \cdot N\mathrm{T}$$

(53)

Once activated, T-cells derived from naïve T-cells can either proliferate or differentiate into memory T-cells. This process is regulated by the function E_N, which is adapted from Bell's paper H function[16]:

$$E_N^{mDC} = \frac{mDC_{tot}}{mDC_{tot} + NT + AT_N + AT_M + MT} \cdot \frac{N_{mDC} - K_{NT}}{N_{mDC} + K_{NT}}$$

(54)

$$E_N^{pDC} = \frac{pDC_{tot}}{pDC_{tot} + NT + AT_N + AT_M + MT} \cdot \frac{N_{pDC} - K_{NT}}{N_{pDC} + K_{NT}}$$

(55)

$$E_N = E_N^{mDC} \cdot w_{mDC} + E_N^{pDC} \cdot w_{pDC}$$

(56)

Where N_{DC} represents the average number of MHC II-antigen complexes presented on the DC plasma membrane, as defined in equations *(46)* and *(47)*.

Finally, we model the population of active T-cells derived from naïve T-cells using the following equation:

$$\frac{dAT_N}{dt} = k_{act}^{NT} \cdot D_N \cdot NT + k_{prol}^{AT} \cdot E_N \cdot AT_N - k_{dt}^{AT} \cdot AT_N$$

(57)

T-cells equation at the draining lymph node - memory T-cell activation equations

When modeling the activation of memory T-cells, it is important to account for the fact that activating a naïve T-cell requires fewer complexes than activating a memory T-cell. To capture this distinction, we introduce a separate parameter, K_{MT}, into the calculation of the DC contribution to memory T-cell activation. In equations *(58)* and *(59)*, we replace the parameter of K_{NT} with K_{MT}, which is set to a value ten times smaller than K_{NT}. This adjustment reflects the reduced number of T-epitope-MHC II-peptide complexes on the DC membrane needed to achieve a 50% activation rate for memory T-cells compared to naïve helper T-cell.

$$D_M^{mDC} = \frac{N_{mDC} \cdot \frac{mDC_{tot}}{mDC_{tot} + NT + AT_N + AT_M + MT}}{N_{mDC} + K_{MT}}$$

(58)

$$D_M^{pDC} = \frac{N_{pDC} \cdot \frac{pDC_{tot}}{pDC_{tot} + NT + AT_N + AT_M + MT}}{N_{pDC} + K_{MT}}$$

(469)

$$D_M = D_M^{mDC} \cdot w_{mDC} + D_M^{pDC} \cdot w_{pDC}$$

(60)

Moreover, we use K_{MT} , instead of K_{NT} as in equations *(54)* and *(55)*, to compute the proliferation/differentiation function E_M, which is adapted from the H function in Bell's paper[16]:

$$E_M^{mDC} = \frac{mDC_{tot}}{mDC_{tot} + NT + AT_N + AT_M + MT} \cdot \frac{N_{mDC} - K_{MT}}{N_{mDC} + K_{MT}}$$

(61)

$$E_M^{pDC} = \frac{pDC_{tot}}{pDC_{tot} + NT + AT_N + AT_M + MT} \cdot \frac{\mathrm{N_{pDC}} - \mathrm{K_{MT}}}{\mathrm{N_{pDC}} + \mathrm{K_{MT}}}$$

(62)

$$E_M = E_M^{mDC} \cdot w_{mDC} + E_M^{pDC} \cdot w_{pDC}$$

(63)

Memory T-cells can originate either from activated naïve T-cells or from proliferating activated memory T-cells. Consequently, in the ordinary differential equation, we include two distinct source terms to account for these processes. Additionally, two negative terms are included to represent the activation of memory T-cells and their potential death.

$$\frac{dMT}{dt} = k_{prol}^{AT} \cdot (1 - E_N) \cdot f^{AT} \cdot AT_N + k_{prol}^{AT} \cdot (1 - E_M) \cdot f^{AT} \cdot AT_M - k_{act}^{MT} \cdot D_M \cdot M\mathrm{T} - k_{dt}^{MT} \cdot M\mathrm{T}$$

(64)

Active T-cells derived from memory T-cells can either proliferate to generate more memory T-cells, or differentiate into functional T-helper cells (FT).

$$\frac{dAT_M}{dt} = k_{act}^{MT} \cdot D_M \cdot M\mathrm{T} + k_{prol}^{AT} \cdot E_M \cdot AT_M - k_{dt}^{AT} \cdot AT_M$$

(65)

$$\frac{dFT}{dt} = k_{prol}^{AT} \cdot (1 - E_N) \cdot (1 - f^{AT}) \cdot AT_N + k_{prol}^{AT} \cdot (1 - E_M) \cdot (1 - f^{AT}) \cdot AT_M - k_{dt}^{FT} \cdot F\mathrm{T}$$

(66)

where f^{AT} is the differentiation percentage of activated helper T-cells that become memory T-cells.

Antigen computation at the draining lymph node

We estimated the total antigen production in the draining lymph node in picomoles (pmol) using the DC maturation weights p_L, p_M, p_H and the maximum number of MHC II-Antigen (MHC II-Ag) complexes displayed on the plasma membrane, as assessed by the molecular layer. Additionally, we employed also the parameter $\exp_{Ag}$ to scale the antigens produced at a localized injection site to a broader immune response in the lymph nodes.

$$\begin{aligned}\mathrm{Ag} = \max_{\mathrm{mDC}}\mathrm{MHCIIAg} \cdot \exp_{Ag} \\ \cdot \Bigg[\Bigg(\Bigg(\left(\frac{(|p_L - p_M|)}{2} + p_L\right) \cdot \left(mDC_{LN}^{Ag}L_{on} + mDC_{LN}^{Ag}L_{off}\right) + \left(\frac{(|p_M - p_H|)}{2} + p_M\right) \\ \cdot \left(mDC_{LN}^{Ag}M_{on} + mDC_{LN}^{Ag}M_{off}\right) + \left(\frac{(|p_H - 1|)}{2} + p_H\right) \cdot \left(mDC_{LN}^{Ag}H\right)\Bigg)\Bigg) \\ + \Bigg(\left(\frac{(|\mathrm{p_L} - \mathrm{p_M}|)}{2} + \mathrm{p_L}\right) \cdot \left(\mathrm{pDC_{LN}^{Ag}L_{on}} + \mathrm{pDC_{LN}^{Ag}L_{off}}\right) + \left(\frac{(|\mathrm{p_M} - \mathrm{p_H}|)}{2} + \mathrm{p_M}\right) \\ \cdot \left(\mathrm{pDC_{LN}^{Ag}M_{on}} + \mathrm{pDC_{LN}^{Ag}M_{off}}\right) + \left(\frac{(|\mathrm{p_H} - 1|)}{2} + \mathrm{p_H}\right) \cdot \left(\mathrm{pDC_{LN}^{Ag}H}\right)\Bigg)\Bigg]\end{aligned}$$

(67)

B-cells equation at the draining lymph node

To model B-cell activation, we primarily reference the work of Chen *et al.* (2014)[4] and Selvaggio *et al.* (2021)[17]. We consider 17 distinct subgroups of B-cells, each characterized by binding affinities $K_{a,i}$ with the antigen

that increase a two-fold between successive subgroups. Consequently, we develop a separate set of ODEs for each clonal population of B-cells. The B-cell receptors (BCRs) also exhibit varying binding affinities, which are calculated as follows:

$$\mathrm{BC}R_i = \mathrm{BRN} \cdot (NB + ANB_i + ANB2_i + MB_i + AMB_i) \cdot \frac{10^{12}}{N_A}$$

(68)

The available antigen for B-cells activation is, then, obtained as the solution of the following equation:

$$\frac{Ag}{V_L N} = Ag_f \cdot \left(1 + \sum_i \frac{K_{a,i} \cdot \frac{BCR_i}{V_{LN}}}{1 + K_{a,i} \cdot Ag_f} \right)$$

(69)

The receptor occupancy $r_{o,i}$ gives a measure of the number of BCR-Ag complexes R_i:

$$r_{o,i} = \frac{K_{a,i} \cdot Ag_f}{1 + K_{a,i} \cdot Ag_f}$$

(70)

$$R_i = r_{o,i} \cdot BCR_i$$

(71)

We can use this information to build a B-cell saturable activation function:

$$\mathrm{F_i} = \frac{R_i}{K_R + R_i}$$

(72)

$$G_i = F_i \cdot \left(1 - r_{o,i}\right)$$

(47)

where K_R is the number of occupied BRCs to achieve a 50% activation rate of naïve B-cells. The function G_i accounts for the tolerance to high antigen dosing. When BCRs become saturated, meaning they are fully bound by antigen, $r_{o,i}$ tends to 1 causing G_i to be null. This models B-cell apoptosis in cases of excessive stimulation by the antigen, which can trigger a cascade of intracellular signals leading to cell death. This mechanism serves to regulate the immune response and eliminate B-cells that become overly active or potentially harmful to the body.

Next, we define the function H_i, which governs the balance between differentiation and proliferation in activated B-cell[4,17] s:

$$H_i = \frac{R_i - K_R}{R_i + K_R}$$

(74)

We introduce the functions P_N and P_M, which model the functional contribution of T-cells to the activation, proliferation and differentiation of naïve and memory B-cell, respectively:

$$P_N = \frac{CC_N \cdot FT}{CC_N \cdot FT + \sum_i NB_i + \sum_i ANB_i + \sum_i GCB_i + \sum_i AMB_i + \sum_i MB_i}$$

(75)

$$P_M = \frac{CC_M \cdot FT}{CC_M \cdot FT + \sum_i NB_i + \sum_i ANB_i + \sum_i GCB_i + \sum_i AMB_i + \sum_i MB_i}$$

(76)

where CC_N and CC_M represent the carrying capacities for functional T-cells to stimulate the activation and proliferation of naïve and memory B-cells, respectively.

Finally, the following set of ODEs describes the B-cell immune response within the draining lymph-node. Inside the draining lymph node, naïve B-cells (*NB*) come into contact with APCs, which present the antigen on their plasma membrane, as well as with functional T-cells (*FT*). This interaction is critical for B-cell activation and maturation. The activation of B-cells (*ANB*) leads to the formation of germinal centers, where activated B-cells at mature stage (*GCB*) and activated B-cells derived from memory B cells (*AMB*), both subclones of activated B-cells, can either proliferate or differentiate in memory B-cells (*MB*) or antibody secreting short-living (*SP)* or long-living (*LP*) plasma cells with varying binding affinities*.

$$\frac{dNB_i}{dt} = k_{dt}^{NB} \cdot (NB0 - NB_i) - k_{act}^{NB} \cdot F_i \cdot P_N \cdot NB_i$$

(77)

$$\frac{dANB_i}{dt} = k_{act}^{NB_i} \cdot G_i \cdot P_N \cdot NB_i - k_{del}^{ANB} \cdot ANB_i - k_{dt}^{AB} \cdot ANB_i$$

(78)

$$\frac{dGCB_i}{dt} = k_{del}^{NB_i} \cdot \mathrm{ANB}_i + k_{prol}^{ANB} \cdot H_i \cdot P_N \cdot GCB_i - k_{dt}^{AB} \cdot GCB_i$$

(79)

$$\frac{dMB_i}{dt} = k_{prol}^{ANB_i} \cdot (1 - H_i) \cdot P_N \cdot g_1 \cdot GCB_i + k_{prol}^{AMB_i} \cdot (1 - H_i) \cdot P_M \cdot g_1 \cdot AMB_i - k_{act}^{MB} \cdot F_i \cdot P_M \cdot MB_i - k_{dt}^{MB} \cdot MB_i$$

(80)

$$\frac{dAMB_i}{dt} = k_{act}^{MB} \cdot G_i \cdot P_M \cdot MB_i + k_{prol}^{AMB} \cdot H_i \cdot P_M \cdot AMB_i - k_{dt}^{AB} \cdot AMB_i$$

(81)

$$\frac{dSP_i}{dt} = k_{prol}^{ANB} \cdot (1 - H_i) \cdot P_N \cdot g_2 \cdot GCB_i + k_{prol}^{AMB} \cdot (1 - H_i) \cdot P_M \cdot g_2 \cdot AMB_i - k_{LN2BL}^{PC} \cdot SP_i - k_{dt}^{SP} \cdot SP_i$$

(82)

$$\frac{dLP_i}{dt} = k_{prol}^{ANB} \cdot (1 - H_i) \cdot P_N \cdot (1 - g_1 - g_2) \cdot GCB_i + k_{prol}^{AMB} \cdot (1 - H_i) \cdot P_M \cdot (1 - g_1 - g_2) \cdot AMB_i - k_{LN2BL}^{PC} \cdot LP_i - k_{dt}^{LP} \cdot LP_i$$

(83)

* Please note that in the code provided in https://github.com/cosbi-research/QSPmRNAVaccines, AMB and ANB can be found as variables named “AB_M” and “AB_N”, respectively.

Where g_1 and g_2 are the percentages of activated B-cells that differentiate into memory B-cells or short-living plasma cells, respectively.

S1.4.3 Blood – BL

In the followings, the generation rates k_{br}^{cells} account for physiological recruitment at steady state under the homeostatic assumption:

$$k_{br}^{cells} = -\mathrm{cells0}_{IS} \cdot k_{IS2BL}^{cells} + \mathrm{cells0}_{BL} \cdot k_{dt}^{Icells}$$

(84)

Where $cells$ refer to any cell population, $cells0_{IS}$ and $cells0_{BL}$ are the initial quantities of naïve cells at the injection site and in the blood, respectively. k_{dt}^{Icells} represents the death rate of immature (naïve) cells in that population, while k_{IS2BL}^{cells} is the migration rate from the injection site to the blood.

Naïve antigen-presenting cells equations in blood

The equations describe the innate immune system, specifically the population of naïve APCs in the blood, which are recruited to the injection site following mRNA vaccine administration.

$$\frac{dNP_{BL}}{dt} = k_{br}^{NP_{BL}} + k_{IS2BL}^{NP} \cdot NP_{IS} - k_{rc}^{NP} \cdot \frac{mRNA_{IS}}{K_{mRNA} + mRNA_{IS}} \cdot NP_{BL} - k_{dt}^{NP} \cdot NP_{BL}$$

(84)

$$\frac{dMN_{BL}}{dt} = k_{br}^{MN_{BL}} + k_{IS2BL}^{MN} \cdot MN_{IS} - k_{rc}^{MN} \cdot \frac{mRNA_{IS}}{K_{mRNA} + mRNA_{IS}} \cdot MN_{BL} - k_{dt}^{MN} \cdot MN_{BL}$$

(85)

$$\frac{dmDC_{BL}}{dt} = k_{br}^{mDC_{BL}} + k_{IS2BL}^{mDC} \cdot mDC_{IS} - k_{rc}^{mDC} \cdot \frac{mRNA_{IS}}{K_{mRNA} + mRNA_{IS}} \cdot mDC_{BL} - k_{dt}^{ImDC} \cdot mDC_{BL}$$

(86)

$$\frac{dpDC_{BL}}{dt} = k_{br}^{pDC_{BL}} + k_{IS2BL}^{pDC} \cdot \mathrm{pDC}_{IS} - k_{rc}^{pDC} \cdot \frac{mRNA_{IS}}{K_{mRNA} + mRNA_{IS}} \cdot \mathrm{pDC}_{BL} - k_{dt}^{IpDC} \cdot \mathrm{pDC}_{BL}$$

(87)

Plasma-cells equations in blood

The equations describe plasma cells migration from the draining lymph node to the blood.

$$\frac{dSP_{BL}}{dt} = k_{LN2BL}^{PC} \cdot SP_{LN} - k_{dt}^{SP} \cdot SP_{BL}$$

(88)

$$\frac{dLP_{BL}}{dt} = k_{LN2BL}^{PC} \cdot LP_{LN} - k_{dt}^{LP} \cdot LP_{BL}$$

(89)

Antibodies equation in blood

The equations describe antibodies secretion in the blood by both short and long-living plasma cells. Antibody production is expressed in picomoles.

$$\frac{dAb_{BL}}{dt} = k_{br}^{Ab} \cdot \frac{SP_{BL}}{N_A \cdot 10^{-12}} += k_{br}^{Ab} \cdot \frac{LP_{BL}}{N_A \cdot 10^{-12}} - k_{dt}^{Ab} \cdot Ab_{BL}$$

(90)

S2. MOLECULAR LAYER FOR MRNA VACCINES

For the molecular layer of the model, we refer to the previous literature[4,18]. We present the tables of model variables (**Section S2.1**), initial values (**Section S2.2S1.S2.2**), parameter estimates (**Section S2.3**), and model equations (**Section S2.4**). The connection between the molecular and cellular layers is explained in **Section S3**.

S2.1 MODEL VARIABLES

Variable name	Variable description	Units
$mRNA_e$	mRNA concentration in the endosome	M
$mRNA_c$	mRNA concentration in the cytosol	M
**Ag**	Antigen	M
**P**	Free-peptide concentration	M
MHC_{PM}^{un}	Unbound MHC on plasma membrane	#molecules
MHC_{INT}^{un}	Unbound MHC internalized by the cell	#molecules
MHC_{PM}^{b}	MHC-peptides on plasma membrane	#molecules
MHC_{INT}^{b}	MHC-peptides complexes internalized by the cell	#molecules

Table S4: *variables of the molecular layer of the model.*

S2.2 INITIAL VALUES

Variable name	description	Initial value	Units	reference
$mRNA0_e$	initial mRNA concentration in the endosome	2.0757e-07	M	*
$mRNA0_c$	initial mRNA concentration in the cytosol	0	M	-
**Ag0**	initial amount of antigen	0	M	-
**P0**	initial amount of free-peptide concentration	0	M	-
$MHC0_{PM}^{un}$	initial number of unbound MHC on plasma membrane	2.00e+05	#molecules	19
$MHC0_{INT}^{un}$	initial number of unbound internalized MHC	0	#molecules	-
$MHC0_{PM}^{b}$	initial number of MHC-peptides on plasma membrane	0	#molecules	-
$MHC0_{INT}^{b}$	initial number of internalized MHC-peptide complexes	0	#molecules	-

Table S5: *initial values of the variables of the molecular layer. *calculated as:* $mRNAmol_{max}/(V_e \cdot N_A)$*, using the values reported in* ***Table S6****.*

S2.3 Model parameters

Parameter	Description	Estimate	Units	Reference
$mRNAmol_{max}$	maximum number of mRNA molecules per LNP	5	pure number	[20]
k_{esc}	mRNA endosomal escape rate	8.3333e-06	L/min	[21]
k_p	antigen production rate via mRNA translation rate	15.72	term/min	computed to target BNT162b2 mRNA sequence, see equation (92)
Ag_{MW}	single strand molecular weight of BNT162b2 mRNA sequence	1.3775e+06	g/mol	Computed with Cusabio molecular weight calculator[11]
k_{dmRNA}	mRNA degradation rate (half-life 10 hours)	0.0012	min^{-1}	[3]
k_d	Dissociation constant for MHC II-peptide complexes	4.2000e-04	min^{-1}	[22]
k_a	Association constant for MHC II-peptide complexes	100	$M^{-1}min^{-1}$	[22]
k_{in}	Rate constant for the internalization of MHC II	1.00E-02	min^{-1}	[23]
k_{out}	Rate constant for vesicle recycle when MHC II are presented on cell membrane	2.00E-02	min^{-1}	[24]
k_{dAg}	Rate constant for degradation of native antigen	1.00E-02	min^{-1}	[25]
k_{syn}	Rate constant for MHC II synthesis	1.10E-03	min^{-1}	[24]
V_c	Cell volume	1.00E-15	L	[26]
V_e	Endosomal volume	4.00E-17	L	[27]
k_{pept}	Rate constant for routing the antigen to lysosomes	1.20E-02	min^{-1}	[22]

Table S6: *model parameters (fitted or retrieved from literature) of the molecular layer.*

S2.4 Model equations

Once the LNPs are internalized by the cell in an endosome, they are degraded, allowing the mRNA to be released into the endosome, from which it subsequently escapes into the cell cytosol.

$$\frac{dmRNA_e}{dt} = -\frac{k_{esc}}{V_e} \cdot \mathrm{mRNA}_e \tag{91}$$

The following equation describes the mRNA released in the cell cytosol and its degradation.

$$\frac{dmRNA_c}{dt} = \frac{k_{esc}}{V_c} \cdot \mathrm{mRNA_e} - k_{dmRNA} \cdot mRNA_c \tag{92}$$

Once the mRNA escapes from the endosome, it is translated by ribosomes into antigen protein. The protein synthesis rate k_p is a sequence-specific parameter evaluated on the BNT162b2 vaccine. It was estimated based on the assumption that the protein synthesis was elongation-limited, and using published data on tRNA abundance in HEK293 cells[28] to model codon decoding times, as described in Chu *et al.* (2014)[29]. This calculation was made when protein synthesis was in the steady-state and assuming that no mRNA degradation occurred during the simulation.

$$\frac{dAg}{dt} = k_p \cdot \frac{V_c}{\mathrm{V_e}} \cdot m\mathrm{RNA}_c - \left(k_{dAg} + k_{pept}\right) \cdot A\mathrm{g}$$

(93)

The produced antigen proteins are degraded into peptides through proteolysis. These free peptides become available for binding to the major histocompatibility complexes of class II (*MHC II*), which are initially internalized in the endosome. The association constant k_a models this binding between the internalized MHC II complexes and free peptides, resulting from the degradation of internalized MHC II-Ag complexes. Conversely, MHC II-Ag complexes can also degrade; in the peptide equation, this is modelled using the dissociation constant k_d.

$$\frac{dP}{dt} = k_{pept} \cdot A\mathrm{g} + \frac{k_d}{V_e} \cdot \frac{M\mathrm{HC}_{INT}^{b}}{N_A} - \frac{k_a}{V_e} \cdot \frac{M\mathrm{HC}_{INT}^{un}}{N_A} \cdot P - k_{dAg} \cdot P$$

(94)

The unbound MHC II complexes on the plasma membrane (MHC_{PM}^{u}) are derived from internalized complexes that are recycled to the cell surface at an externalization rate k_{out}. Conversely, these complexes can also be internalized at a rate k_{in}. Under steady-state conditions and the homeostatic assumption, we assume that they degrade at the same rates as their synthesis, denoted as k_{syn}.

$$\frac{dMHC_{PM}^{un}}{dt} = k_{out} \cdot MHC_{INT}^{un} - k_{in} \cdot MHC_{PM}^{un} + k_d \cdot MHC_{PM}^{b} - k_{syn} \cdot MHC_{PM}^{un}$$

(95)

The unbound internalized MHC II complexes (MHC_{INT}^{un}) can originate from several sources. First, they may arise from the internalization of unbound complexes on the plasma membrane. Second, they can result from the dissociation of internalized bound MHC II-P complexes. Lastly, new complexes can be synthesized within the cell. The loss terms in the equation MHC_{INT}^{un} are attributed to the association with free peptides or the externalization of the unbound complexes.

$$\frac{dMHC_{INT}^{un}}{dt} = k_{in} \cdot MHC_{PM}^{un} - k_{out} \cdot MHC_{INT}^{un} + k_d \cdot MHC_{INT}^{b} - k_a \cdot MHC_{INT}^{un} \cdot P + k_{syn} \cdot \left(MHC_{PM}^{un} + MHC_{PM}^{b}\right)$$

(96)

Bound internalized MHC II-antigen peptide complexes (MHC_{INT}^{b}) arise from the association between free peptides and unbound complexes. Once bound, MHC II-P complexes can either be degraded or move to the plasma membrane.

$$\frac{dMHC_{INT}^{b}}{dt} = k_a \cdot MHC_{INT}^{un} \cdot P - k_d \cdot MHC_{INT}^{b} - k_{out} \cdot MHC_{INT}^{b}$$

(97)

Bound MHC II-antigen peptide complexes on the plasma membrane (MHC_{PM}^{b}) have two loss terms: one represents the dissociation between the MHC II complexes and the peptides, while the other accounts for the degradation of the complexes.

$$\frac{dMHC_{PM}^{b}}{dt} = k_{\text{out}} \cdot M\text{HC}_{INT}^{b} - k_{syn} \cdot M\text{HC}_{PM}^{b} - k_{d} \cdot M\text{HC}_{PM}^{b} \tag{98}$$

S3. MOLECULAR AND TISSUE LAYERS CONNECTION

To bridge the gap between the molecular layer and the tissue structure of the model, we developed an optimization procedure for the dendritic cell maturation rates. This allows us to utilize information derived from the molecular layer to inform the maturation dynamics of dendritic cells.

From the molecular layer, we obtain the curve representing the exposure of MHC II-Ag complexes on the plasma membrane, from which we define three different exposure weights: low ($w_L = 0.01$), medium ($w_M = 0.4$), and high ($w_H = 0.9$). By applying these weights to the maximum of the exposure curve, we establish three exposure thresholds:

$$T_L = w_L \cdot \text{max}_{\text{DC}}\text{MHCIIAg},$$
$$T_M = w_M \cdot \text{max}_{\text{DC}}\text{MHCIIAg},$$
$$T_H = w_H \cdot \text{max}_{\text{DC}}\text{MHCIIAg}. \tag{99}$$

Given these exposure thresholds, we identify the intersection points between the curve and the thresholds:

- M_{tr} is the intersection between the curve and T_M during the binding phase,
- H_{tr} is the intersection between the curve and T_H during the binding phase,
- H_{atr} is the intersection between the curve and T_H during the unbinding phase,
- M_{atr} is the intersection between the curve and T_M during the unbinding phase,
- L_{atr} is the intersection between the curve and T_L during the unbinding phase.

These points define five different time intervals on the x-axis: Δt_1, $\Delta t_2, \Delta t_3, \Delta t_4, \Delta t_5$.

The goal is to align these time intervals with the mean residence time of maturing dendritic cells. To achieve this, we implement a more parsimonious version of the cellular scale, focusing solely on the dendritic maturation process within a general compartment, under the assumption that maturation rates remain consistent between the injection site and the draining lymph node. We then evaluate the mean residence times within this simplified maturation chain and estimate the maturation rates to match the mean residence times with the exposure time intervals (Δt_1,…, Δt_5) of the molecular layer (Figure S1).

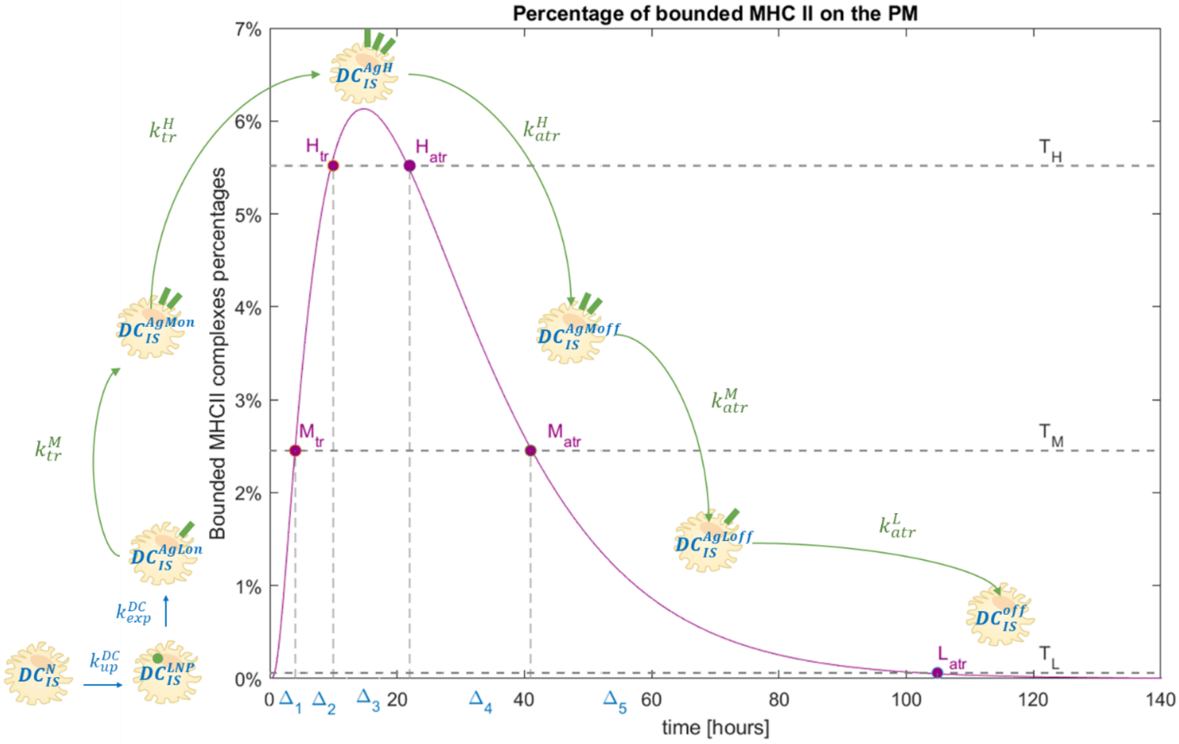

Figure *S*1: Molecular and *tissue* scale connection. *The mean residence time of the maturing dendritic cells is matched with the time intervals ($\Delta_1, ..., \Delta_5$) identified on the MHC II-Ag exposure curve by the thresholds T_L, T_M and T_H. In this way we estimate the maturation rates of dendritic cells. Created in BioRender. Marchetti, L. (2025) https://BioRender.com/w16b529.*

This approach allows us to incorporate molecular information, such as the mRNA translation velocity, into our model. These product-specific details are crucial in tailoring our simulations to the specific product under consideration.

S4. ANTIBODY LEVEL EXPERIMENTAL DATA AND HARMONIZATION OF DIFFERENT MEASURE UNITS

The time dynamics of plasma antibody levels following COVID-19 vaccination have been extensively studied, with various assays and units of measurement employed. As Muller *et al.* (2022)[30] demonstrated, while results from different studies are correlated, they are not always directly comparable. Since our model describes on the dynamics of anti-RBD IgG concentrations in plasma, we utilized data from Keshavarz *et al.* (2022)[8] and Goel *et al.* (2021)[9], which report the concentration of anti-RBD IgG in plasma following the administration of the BNT162b2 and mRNA-1273 vaccines.

For the calibration of our model using the BNT162b2 vaccine, we also incorporated clinical trial data[10], which tested four different doses: 1 µg, 10 µg, 20 µg, and 30 µg. The data from the two lowest and highest doses were used for calibration, while the intermediate doses were used for validation. The clinical trial data, originally expressed in U/mL, were rescaled to align with plasma concentration data. The scaling factor was estimated by comparing the data from Keshavarz *et al.* (2022)[8] and Goel *et al.* (2021)[9] with the U/mL data

from corresponding dose experiments in the clinical trial. To ensure comparability at common time points, a moving average of the concentration data was computed using a 6-day window. This same approach was applied to identify the scaling factor for additional data used in model validation[31–33] (**Figure 4c**), which were expressed in AU/mL using the ARCHITECT® assay. We used the same procedure for the data from Payne et al. (2021)[34] and for the data from Roltgen *et al.* (2022)[35].

Reference	Measured variables (unit of measure)	Assay	mRNA vaccine	Dose and scheduling	Study type
Liang *et al.*, 2014[2]	NPs, MNs, mDCs, pDCs at IS (#cells/g) and LN (#cells)	Rockland HAI assay	anti-H10N8 influenza virus	One dose - 50µg	Preclinical (Rhesus macaques)
Keshavarz *et al.*, 2022[8]	RBD IgG (ng/mL)	ImmunoCAP-based assay	Pfizer–BioNTech BNT162b2	Two doses (day 0, 21) - 30µg	Clinical study
Keshavarz *et al.*, 2022[8]	RBD IgG (ng/mL)	ImmunoCAP-based assay	Moderna mRNA-1273	Two doses (day 0, 28) - 100µg	Clinical study
Goel *et al.*, 2021[9]	RBD IgG (µg/mL)	modified ELISA assay[36]	Pfizer–BioNTech BNT162b2	Two doses (day 0, 21) - 30µg	Clinical study
Sahin *et al.*, 2021[10]	RBD IgG (U/mL)	Acro Biosystems, SPD-C82E9	Pfizer–BioNTech BNT162b2	Two doses (day 0, 21) - 1µg, 10µg, 20µg, 30µg	Phase-I/II clinical trial
Jochum *et al.*, 2022[37]	RBD IgG (U/mL)	Elecsys® ACOV2S	Moderna mRNA-1273	Two doses (day 0, 28) - 25µg , 100µg	Phase-I clinical trial
Kirste *et al.*, 2023[38]	RBD IgG (U/mL)	Elecsys® ACOV2S	Moderna mRNA-1273	Two doses (day 0, 28) – 50µg, 100µg	Phase-II clinical trial
Naaber *et al.*, 2021[31]	RBD IgG (AU/mL)	ARCHITECT®	Pfizer–BioNTech BNT162b2	Two doses (day 0, 21) - 30µg	Clinical study
Kontopoulou *et al.*, 2022[33]	RBD IgG (AU/mL)	ARCHITECT®	Pfizer–BioNTech BNT162b2	Three doses (day 0, 21, 291) - 30µg	Clinical study
Takeuchi *et al.*, 2022[32]	RBD IgG (AU/mL)	ARCHITECT®	Pfizer–BioNTech BNT162b2	Two doses (day 0, 21) - 30µg	Clinical study
Payne *et al.*, 2021 [34]	RBD IgG (AU/mL)	multiplexed MSD immunoassay	Pfizer–BioNTech BNT162b2	Two doses (day 0, 70) - 30µg	Clinical study
Roltgen *et al.*, 2022[35]	RBD IgG (AU/mL)	V-PLEX® COVID-19 Coronavirus Panel 2	Pfizer–BioNTech BNT162b2	Two doses (day 0, 21) - 30µg	Clinical study

Table S7: *list of data sources used for calibration and/or validation of the model, with indications on measured variables, unit of measure, assays, vaccine type, protocol and study type (clinical or preclinical).*

For the validation of the parameterization of the model for the mRNA-1273 vaccine, we utilized clinical trial data [37,38]. These trials tested different doses—25 µg and 50 µg—alongside the 100 µg dose, which was also reported by Keshavarz *et al.* (2022)[8] and Goel *et al.* (2021)[9]. Antibody levels were measured using the Roche Elecsys® Anti-SARS-CoV-2 S assay (ACOV2S). To rescale the antibody levels reported for the 25 µg and 50 µg dose regimens, we relied on Section 3.2 and on Figure 3A of Jochum *et al*. (2022)[37] , where the authors show a linear correlation between the logarithm of the measurements by ACOV2S and the logarithm of the ones

by RBD ELISA. We therefore fitted a nonlinear regression model to the 100 µg dose data from Jochum *et al*. (2022)[37] and the 100µg dose data from Keshavarz at al. (2022)[8]. With this model we finally projected the clinical trial data for the 25 µg and 50 µg doses.

Reference	mRNA vaccine	Dose and scheduling	Sample size
Liang *et al.*, 2014[2]	anti-H10N8 influenza virus	One dose - 50µg	5 subjects (10 measurements) at the timepoints 0h, 4h, 24h, and 2 subjects (2 measurements) at the timepoint 9d
Keshavarz *et al.*, 2022[8]	Pfizer–BioNTech BNT162b2	Two doses (day 0, 21) - 30µg	114 subjects
Keshavarz *et al.*, 2022[8]	Moderna mRNA-1273	Two doses (day 0, 28) - 100µg	114 subjects
Goel *et al.*, 2021[9]	Pfizer–BioNTech BNT162b2	Two doses (day 0, 21) - 30µg	33 subjects (3 over-60 subjects)
Sahin *et al.*, 2021[10]	Pfizer–BioNTech BNT162b2	Two doses (day 0, 21) - 1µg, 10µg, 20µg, 30µg	• 1µg: 12 subjects at the timepoints 1d, 8±1d, 22 ±2d, 11 subjects at the timepoint 29±3d, 10 subjects at the timepoints 43±4d, 50±4d<br>• 10µg: 12 subjects at the timepoints 1d, 8±1d, 11 subjects at the timepoints 22 ±2d, 29±3d, 43±4d, 50±4d, 85±7d<br>• 20µg: 12 subjects at the timepoints 1d, 8±1d, 22 ±2d, 29±3d, 43±4d, 50±4d, 10 subjects at the timepoint 85±7d<br>• 30µg: 12 subjects at the timepoints 1d, 8±1d, 22 ±2d, 29±3d, 50±4d, 85±7d, 11 subjects at the timepoint 43±4d
Jochum *et al.*, 2022[37]	Moderna mRNA-1273	Two doses (day 0, 28) - 25µg , 100µg	• 25µg: 15 subjects at all the timepoints<br>• 100µg: 15 subjects at the timepoints 1d, 15d, 29d, 14 subjects at the timepoints 43d and 57d
Kirste *et al.*, 2023[38]	Moderna mRNA-1273	Two doses (day 0, 28) – 50µg, 100µg	• 50µg: 197 subjects<br>• 100µg: 198 subjects
Naaber *et al.*, 2021[31]	Pfizer–BioNTech BNT162b2	Two doses (day 0, 21) - 30µg	88 subjects at 0d, 111 subjects at 21d, 106 subjects at 28d, 89 subjects at 63d, 90 subjects at 105d and 201d
Kontopoulou *et al.*, 2022[33]	Pfizer–BioNTech BNT162b2	Three doses (day 0, 21, 291) - 30µg	111 subjects (19 over-60 subjects)
Takeuchi *et al.*, 2022[32]	Pfizer–BioNTech BNT162b2	Two doses (day 0, 21) - 30µg	100 subjects
Payne *et al.*, 2021 [34]	Pfizer–BioNTech BNT162b2	Two doses (day 0, 70) - 30µg	94 subjects
Roltgen *et al.*, 2022[35]	Pfizer–BioNTech BNT162b2	Two doses (day 0, 21) - 30µg	5 over-60 subjects

Table S8: *list of data sources used for calibration and/or validation of the model, with indications on the sample size.*

S5. Calibration procedure

For the calibration of model parameters, we adopted a multistep workflow, detailed as follows.

Step 1: calibration of the parameters for early events. To calibrate the parameters describing the early events following mRNA vaccine administration, we focused on the tissue layer representing four populations of APCs (neutrophils, monocytes, myeloid and plasmacytoid dendritic cells) at the injection site and within the lymph node. Additionally, we considered dendritic cells prior to antigen expression (equations 10, 18, 29 and 36) without specifying expression levels. Calibration was based on data from Liang *et al.* (2017)[2], used to estimate 29 parameters (**Table S3**). The model was calibrated by maximizing the likelihood, assuming a proportional error model. Before fitting, the *a priori* global structural identifiability of model parameters was assessed using the GenSSI software toolbox[39]. Data were extracted from the original plots reported in the paper from Liang *et al.* (2017)[2] using the WebPlotDigitizer online tool[40].

Step 2: calibration of dendritic cell maturation rates. After fixing the parameter estimates in Step 1, we developed a more parsimonious model version, focusing solely on the dendritic cell maturation chain in a general compartment. This simplified version, together with the molecular model, was employed to calibrate dendritic cell maturation rates, as described in **Section S3**.

Step 3: calibration of the remaining parameters. The remaining 15 parameters from the tissue layer were calibrated to optimize model predictions against experimental data detailing antibody time dynamics[8–10] (**Table S3**). Calibration was performed by maximizing likelihood, assuming an exponential error model, with the likelihood weighted according to dataset size.

Special calibrations of the model. For calibrations of the model for mRNA-1273 vaccine[8] or stratified patients following BNT162b2 vaccination[9], the parameters from Step 3 were recalibrated (except for the scaling factor $\boldsymbol{exp_{Ag}}$, between the injection site and the lymph node, assumed constant). For patients over 60 years of age, a Bayesian fit was performed, incorporating the L2 norm of the deviation from general population parametrization into the cost function[41]. The parameter estimates for these calibrations are listed in **Table S9**.

Parameter	Description	Unit of measure	Calibration with Moderna data[8]	Calibration with Pfizer over 60 y.o. data[9,33,35]
$\boldsymbol{k_{act}^{NT}}$	Maximum activation rate for NTs	day^{-1}	294.2036	305.5939
$\boldsymbol{k_{act}^{MT}}$	Maximum activation rate for memory T cells	day^{-1}	936.0983	637.5927
$\boldsymbol{k_{prol}^{AT}}$	Maximum proliferation rate for activated T-cells	day^{-1}	4.9806	6.2400
$\boldsymbol{CC_N}$	Carrying capacity of functional T cells to stimulate the activation of naïve B cells		66.9962	96.6608
$\boldsymbol{k_{prol}^{AB_N}}$	Maximum proliferation rate for activated B-cells derived from naïve B cells	day^{-1}	6.0706	9.7873
$\boldsymbol{k_{prol}^{AB_M}}$	Maximum proliferation rate for activated B-cells derived from memory B cells	day^{-1}	6.2593	2.9112

k_{dt}^{SP}	Death rate of short-lived plasma cells	day^{-1}	0.1448	0.0651
k_{dt}^{LP}	Death rate of long-lived plasma cells	day^{-1}	0.0143	0.0070
k_{act}^{MB}	Maximum activation rate for memory B cells	day^{-1}	8.6647	14.2525
k_{LN2BL}^{PC}	Migration rate from LN to BL of plasma cells	day^{-1}	40.2307	23.8881
$delay_B$	Migration rate for the activated B cells to germinal center	day^{-1}	3.7520e-08	8.1200e-04
k_{deg}^{Ab}	Elimination rate of Ab in BL	day^{-1}	0.0931	0.1424

Table S9: *model parameter estimates for the mRNA-1273 vaccine and for the BNT162b2 vaccine (over 60-year-old individuals).*

S6. EXTENDED DOSING INTERVAL OF BNT162B2 VACCINE

As a complement to the discussion in Section 4.3 (Simulation of optimal dosing schedules) of the Main Text, in the figure below we display a comparison between the data from Payne *et al.* (2021)[34], corresponding to the extended interval protocol where a second dose of the BNT162b2 vaccine has been administered 10 weeks after priming, and the threshold of 4422.11 ng/mL, defined as the geometric mean concentration of anti-RBD IgG in convalescent serum, utilized by Giorgi *et al.* (2021)[42] to indicate a protective antibody level.

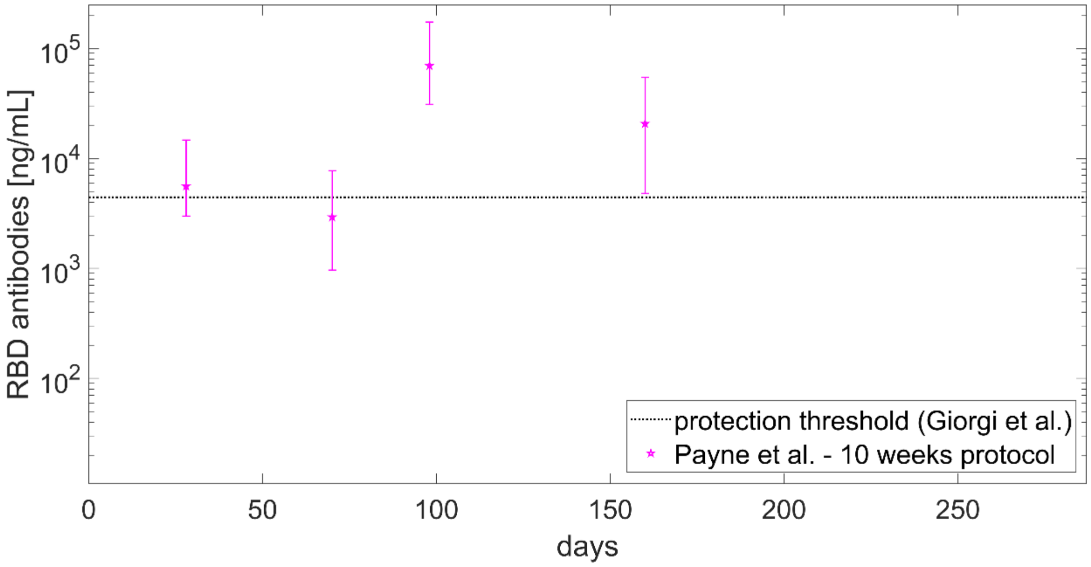

Figure S2: Comparison between data for an extended protocol of BNT162b2 mRNA vaccine and a protective threshold. *Data from Payne et al. (2021)[34] of a two-dose vaccination protocol with BNT162b2, where the second dose has been administered 10 weeks after priming, plotted together with the geometric mean concentration of anti-RBD IgG in convalescent serum from Giorgi et al. (2021)[42]. Stars with error bars represent median values with interquartile range. The plotted data were harmonized using scaling factors derived as explained in* ***Supplementary Section S4****.*

S7. SUPPLEMENTARY DISCUSSION

This section expands on additional points that were excluded from the main discussion due to manuscript length constraints.

S7.1 MODEL EXTENSION TO OTHER VACCINES

The goal of our work is to lay the foundation for a mechanistic platform that enables the direct integration of vaccine-specific attributes. We focused our initial efforts on FDA-approved mRNA vaccines against COVID-19, due to their importance in answering to the pandemic emergence. For this reason, the model has been calibrated using the mRNA-1273 and BNT162b2 vaccines, both of which encode the same antigen protein. Further investigation is required to establish the versatility of our model as a customizable platform for other mRNA vaccines.

The potential to incorporate vaccine-specific attributes into the model is the primary motivation for explicitly describing the dynamics of LNP-mRNA uptake and mRNA translation within antigen-presenting cells in the molecular layer of the model (Supplementary Section S2). This structure enables the possibility of mapping vaccine attributes to specific parameters within the model. However, due to the complex nature of intracellular processes and the multifaceted influence of vaccine properties, not all the attributes can be directly associated with a single model parameter.

We explicitly described the translation process to directly incorporate the translation rate of an mRNA sequence into our model. The current translation rate was *ad hoc* computed considering the mRNA sequence length used in the mRNA-1273 and BNT162b2 vaccines. It was estimated based on the assumption that the protein synthesis was elongation-limited, and using published data on tRNA abundance in HEK293 cells[28] to model codon decoding times, as described in Chu *et al.* (2014)[29]. This calculation was made when protein synthesis was in the steady-state and assuming that no mRNA degradation occurred during the simulation. When calibrating the model for other vaccines, data about the new protein would be required to estimate the translation rate.

Other vaccine attributes—such as the composition of the LNP delivery system— cannot be mapped to a specific molecular-layer parameter. *In silico* prediction of the biological properties of LNPs based on their physicochemical properties is an active area of research[43]. Several machine-learning-based tools have been developed to predict the transfection efficacy of LNPs based on their chemical composition[44,45]. Since transfection efficacy encompasses all processes leading to protein synthesis, these tools can be used in future to scale all processes in the molecular layer contributing to mRNA translation.

S7.2 NOVELTY AGAINST EXISTING IS/ID MODELS

An alternative approach for the description of the immune response to vaccine is represented by semi-mechanistic frameworks such as Immunostimulatory/Immunodynamics (IS/ID) models. Ivaturi *et al.*[46] propose a semi-mechanistic model that explicitly describes the biological processes occurring within the lymph node after the injection of an mRNA-vaccine, while employing a data-driven approach to characterize the earlier immune responses leading up to B-cell activation. Specifically, in a single-dose scenario, B-cell activation over time is modeled via a phenomenological Gaussian function:

$$\delta(t) = a \cdot \exp\left(-\frac{(t-b)^2}{c^2}\right)$$

where a, b and c are estimated parameters derived from available data.

This semi-mechanistic approach has both strengths and limitations compared to a fully mechanistic model. On the one hand, IS/ID models benefit from reduced complexity, allowing for the use of standard pharmacometrics tools to estimate intra- and inter-individual variability and perform covariate analyses[46]. In contrast, the higher complexity of the equations of our mechanistic model poses challenges in this regard. On the other hand, our fully mechanistic approach provides a foundation for key advancements. First, a molecular-level description of intracellular processes can set the basis to directly incorporate specific characteristics of the vaccine product, such as variations in LNP formulation and mRNA translation rates, linking these changes to parameter modifications. In contrast, a data-driven approach would require additional clinical datasets and a novel model calibration for each vaccine, to estimate the parameters of $\delta(t)$. Moreover, a mechanistic framework offers significant advantages in capturing biological inter-individual variability and analyzing specific covariates.

S7.3 Model capability to describe the immune response to virus mutation

Evaluating the effectiveness of an mRNA vaccine against spike variants or predicting the immune response to a booster against new variants *in silico* presents two key interconnected challenges that limit our ability to perform such an analysis.

The first challenge is understanding how variations in the spike protein genome impact the efficacy of a vaccine originally developed for the wild-type virus or an earlier variant. During the COVID-19 pandemic, clinical data remained essential to determine whether existing vaccines were losing effectiveness against emerging variants[47]. In recent years, there have been attempts to evaluate, *in silico*, the loss of antibody binding affinity due to changes in spike protein epitope topology[48]. However, these studies focus on structural biology, operating at a different level of detail compared to our QSP model.

The second key challenge relates to the concept of immune imprinting[49], which refers to the immune system tendency to rely on pre-existing memory responses when encountering a new but related virus variant. This reliance can potentially lead to lower-affinity responses against the new variant. However, there is no clear consensus in the literature regarding the impact of immune imprinting on immune responses to new variants[50]. On the one hand, some studies suggest that immune imprinting may inhibit the emergence of B cells targeting new variants. As a result, imprinting induced by prior vaccination could compromise the antibody response to variant-based boosters, as has been investigated in the context of the Omicron variant of SARS-CoV-2[51,52]. On the other hand, other studies propose that older memory B-cell responses are not misdirected but rather adapt and shift their focus from the previous strain to the new variant[53]. This lack of consensus on the biological mechanisms underlying immune imprinting, particularly when the immune system encounters a booster against a new variant, poses significant challenges in developing a mechanistic model to address this question.

BIBLIOGRAPHY

1. World Health Organization - Interim recommendations for use of the Pfizer–BioNTech COVID-19 vaccine, BNT162b2, under emergency use listing: interim guidance [Internet]. [cited 2024 Jul 7]; Available from: https://iris.who.int/bitstream/handle/10665/338484/WHO-2019-nCoV-vaccines-SAGE_recommendation-BNT162b2-2021.1-eng.pdf?sequence=1&isAllowed=y

2. Liang F, Lindgren G, Lin A, Thompson EA, Ols S, Röhss J, John S, Hassett K, Yuzhakov O, Bahl K, et al. Efficient Targeting and Activation of Antigen-Presenting Cells In Vivo after Modified mRNA Vaccine Administration in Rhesus Macaques. Molecular Therapy 2017; 25:2635–47.

3. Yang E, van Nimwegen E, Zavolan M, Rajewsky N, Schroeder M, Magnasco M, Darnell JE. Decay Rates of Human mRNAs: Correlation With Functional Characteristics and Sequence Attributes. Genome Res 2003; 13:1863–72.

4. Chen X, Hickling TP, Vicini P. A mechanistic, multiscale mathematical model of immunogenicity for therapeutic proteins: Part 1 - Theoretical model. In: CPT: Pharmacometrics and Systems Pharmacology. Nature Publishing Group; 2014.

5. Donners R, Yiin RSZ, Blackledge M, Koh D-M. Whole-body diffusion-weighted MRI of normal lymph nodes: prospective apparent diffusion coefficient histogram and nodal distribution analysis in a healthy cohort. Cancer Imaging 2021; 21:64.

6. Widmaier EP, Vander AJ, Raff H, Strang KT. Human Physiology : the Mechanisms of Body Function. Boston: McGraw-Hill Higher Education; 2004.

7. Tiesinga E, Mohr PJ, Newell DB, Taylor BN. CODATA Recommended Values of the Fundamental Physical Constants: 2018. J Phys Chem Ref Data 2021; 50.

8. Keshavarz B, Richards NE, Workman LJ, Patel J, Muehling LM, Canderan G, Murphy DD, Brovero SG, Ailsworth SM, Eschenbacher WH, et al. Trajectory of IgG to SARS-CoV-2 After Vaccination With BNT162b2 or mRNA-1273 in an Employee Cohort and Comparison With Natural Infection. Front Immunol 2022; 13.

9. Goel RR, Apostolidis SA, Painter MM, Mathew D, Pattekar A, Kuthuru O, Gouma S, Hicks P, Meng W, Rosenfeld AM, et al. Distinct antibody and memory B cell responses in SARS-CoV-2 naïve and recovered individuals after mRNA vaccination [Internet]. 2021. Available from: https://www.science.org

10. Sahin U, Muik A, Vogler I, Derhovanessian E, Kranz LM, Vormehr M, Quandt J, Bidmon N, Ulges A, Baum A, et al. BNT162b2 vaccine induces neutralizing antibodies and poly-specific T cells in humans. Nature 2021; 595:572–7.

11. https://www.cusabio.com/m-299.html.

12. Celli S, Day M, Müller AJ, Molina-Paris C, Lythe G, Bousso P. How many dendritic cells are required to initiate a T-cell response? Blood 2012; 120:3945–8.

13. Korosec CS, Farhang-Sardroodi S, Dick DW, Gholami S, Ghaemi MS, Moyles IR, Craig M, Ooi HK, Heffernan JM. Long-term durability of immune responses to the BNT162b2 and mRNA-1273 vaccines based on dosage, age and sex. Sci Rep 2022; 12.

14. Jones DD, Wilmore JR, Allman D. Cellular Dynamics of Memory B Cell Populations: IgM+ and IgG+ Memory B Cells Persist Indefinitely as Quiescent Cells. The Journal of Immunology 2015; 195:4753–9.

15. Lee HY, Topham DJ, Park SY, Hollenbaugh J, Treanor J, Mosmann TR, Jin X, Ward BM, Miao H, Holden-Wiltse J, et al. Simulation and Prediction of the Adaptive Immune Response to Influenza A Virus Infection. J Virol 2009; 83:7151–65.

16. Bell GI. Mathematical Model of Clonal Selection and Antibody Production. 1970.

17. Selvaggio G, Leonardelli L, Lofano G, Fresnay S, Parolo S, Medini D, Siena E, Marchetti L. A quantitative systems pharmacology approach to support mRNA vaccine development and optimization. CPT Pharmacometrics Syst Pharmacol2021; 10:1448–51.

18. Agrawal NG, Linderman JJ. Mathematical modeling of helper T lymphocyte/antigen-presenting cell interactions: analysis of methods for modifying antigen processing and presentation. J Theor Biol 1996; 182:487–504.

19. Harding C V, Unanue ER. Antigen processing and intracellular Ia. Possible roles of endocytosis and protein synthesis in Ia function. The Journal of Immunology 1989; 142:12–9.

20. Carrasco MJ, Alishetty S, Alameh M-G, Said H, Wright L, Paige M, Soliman O, Weissman D, Cleveland TE, Grishaev A, et al. Ionization and structural properties of mRNA lipid nanoparticles influence expression in intramuscular and intravascular administration. Commun Biol 2021; 4:956.

21. Mihaila R, Ruhela D, Keough E, Cherkaev E, Chang S, Galinski B, Bartz R, Brown D, Howell B, Cunningham JJ. Mathematical Modeling: A Tool for Optimization of Lipid Nanoparticle-Mediated Delivery of siRNA. Mol Ther Nucleic Acids 2017; 7:246–55.

22. Singer DF, Linderman JJ. The relationship between antigen concentration, antigen internalization, and antigenic complexes: modeling insights into antigen processing and presentation. J Cell Biol 1990; 111:55–68.

23. Tse DB, Pernis B. Spontaneous internalization of Class I major histocompatibility complex molecules in T lymphoid cells. J Exp Med 1984; 159:193–207.

24. Tse DB, Cantor CR, McDowell J, Pernis B. Recycling class I MHC antigens: dynamics of internalization, acidification, and ligand-degradation in murine T lymphoblasts. J Mol Cell Immunol 1986; 2:315–29.

25. LAUFFENBURGER DA, LINDERMAN J, BERKOWITZ L. Analysis of Mammalian Cell Growth Factor Receptor Dynamics a. Ann N Y Acad Sci 1987; 506:147–62.

26. Wiśniewski JR, Hein MY, Cox J, Mann M. A "Proteomic Ruler" for Protein Copy Number and Concentration Estimation without Spike-in Standards. Molecular & Cellular Proteomics 2014; 13:3497–506.

27. Marsh M, Griffiths G, Dean GE, Mellman I, Helenius A. Three-dimensional structure of endosomes in BHK-21 cells. Proceedings of the National Academy of Sciences 1986; 83:2899–903.

28. Pavon-Eternod M, Wei M, Pan T, Kleiman L. Profiling non-lysyl tRNAs in HIV-1. RNA 2010; 16:267–73.

29. Chu D, Kazana E, Bellanger N, Singh T, Tuite MF, von der Haar T. Translation elongation can control translation initiation on eukaryotic mRNAs. EMBO J 2014; 33:21–34.

30. Müller L, Kannenberg J, Biemann R, Hönemann M, Ackermann G, Jassoy C. Comparison of the measured values of quantitative SARS-CoV-2 spike antibody assays. Journal of Clinical Virology 2022; 155:105269.

31. Naaber P, Tserel L, Kangro K, Sepp E, Jürjenson V, Adamson A, Haljasmägi L, Rumm AP, Maruste R, Kärner J, et al. Dynamics of antibody response to BNT162b2 vaccine after six months: a longitudinal prospective study. The Lancet Regional Health - Europe 2021; 10.

32. Takeuchi JS, Fukunaga A, Yamamoto S, Tanaka A, Matsuda K, Kimura M, Kamikawa A, Kito Y, Maeda K, Ueda G, et al. SARS-CoV-2 specific T cell and humoral immune responses upon vaccination with BNT162b2: a 9 months longitudinal study. Sci Rep 2022; 12.

33. Kontopoulou K, Nakas CT, Papazisis G. Significant Increase in Antibody Titers after the 3rd Booster Dose of the Pfizer–BioNTech mRNA COVID-19 Vaccine in Healthcare Workers in Greece. Vaccines (Basel) 2022; 10.

34. Payne RP, Longet S, Austin JA, Skelly DT, Dejnirattisai W, Adele S, Meardon N, Faustini S, Al-Taei S, Moore SC, et al. Immunogenicity of standard and extended dosing intervals of BNT162b2 mRNA vaccine. Cell 2021; 184:5699-5714.e11.

35. Röltgen K, Nielsen SCA, Silva O, Younes SF, Zaslavsky M, Costales C, Yang F, Wirz OF, Solis D, Hoh RA, et al. Immune imprinting, breadth of variant recognition, and germinal center response in human SARS-CoV-2 infection and vaccination. Cell 2022; 185:1025-1040.e14.

36. Amanat F, Stadlbauer D, Strohmeier S, Nguyen THO, Chromikova V, McMahon M, Jiang K, Arunkumar GA, Jurczyszak D, Polanco J, et al. A serological assay to detect SARS-CoV-2 seroconversion in humans. Nat Med 2020; 26:1033–6.

37. Jochum S, Kirste I, Hortsch S, Grunert VP, Legault H, Eichenlaub U, Kashlan B, Pajon R. Clinical Utility of Elecsys Anti-SARS-CoV-2 S Assay in COVID-19 Vaccination: An Exploratory Analysis of the mRNA-1273 Phase 1 Trial. Front Immunol 2022; 12.

38. Kirste I, Hortsch S, Grunert VP, Legault H, Maglinao M, Eichenlaub U, Kashlan B, Pajon R, Jochum S. Quantifying the Vaccine-Induced Humoral Immune Response to Spike-Receptor Binding Domain as a Surrogate for Neutralization Testing Following mRNA-1273 (Spikevax) Vaccination Against COVID-19. Infect Dis Ther 2023; 12:177–91.

39. Chiş O, Banga JR, Balsa-Canto E. GenSSI: a software toolbox for structural identifiability analysis of biological models. Bioinformatics 2011; 27:2610–1.

40. https://automeris.io/.

41. Chiuso A. Regularization and Bayesian learning in dynamical systems: Past, present and future. Annu Rev Control 2016; 41:24–38.

42. Giorgi M, Desikan R, van der Graaf PH, Kierzek AM. Application of quantitative systems pharmacology to guide the optimal dosing of COVID-19 vaccines. CPT Pharmacometrics Syst Pharmacol2021; 10:1130–3.

43. Yuan Z, Yan R, Fu Z, Wu T, Ren C. Impact of physicochemical properties on biological effects of lipid nanoparticles: Are they completely safe. Science of The Total Environment 2024; 927:172240.

44. Moayedpour S, Broadbent J, Riahi S, Bailey M, V. Thu H, Dobchev D, Balsubramani A, N.D. Santos R, Kogler-Anele L, Corrochano-Navarro A, et al. Representations of lipid nanoparticles using large language models for transfection efficiency prediction. Bioinformatics 2024; 40.

45. Xu Y, Ma S, Cui H, Chen J, Xu S, Gong F, Golubovic A, Zhou M, Wang KC, Varley A, et al. AGILE platform: a deep learning powered approach to accelerate LNP development for mRNA delivery. Nat Commun 2024; 15:6305.

46. Ivaturi V, Attarwala H, Deng W, Ding B, Schnyder Ghamloush S, Girard B, Iqbal J, Minnikanti S, Zhou H, Miller J, et al. Immunostimulatory/Immunodynamic model of mRNA-1273 to guide pediatric vaccine dose selection. CPT Pharmacometrics Syst Pharmacol 2025; 14:42–51.

47. Han X, Ye Q. The variants of SARS-CoV-2 and the challenges of vaccines. J Med Virol 2022; 94:1366–72.

48. Vissapragada M, Aggunna M, Tallapalli M, Mandugula H, Devandla A, Yekula A, Malapati A, Bonala S, Addala S, Gudapati S, et al. In silico prediction of COVID-19 vaccine efficacy based on the strain-specific structural deviations in the SARS CoV-2 spike protein receptor binding domain. Med Res Arch 2024; 12.

49. Huang CQ, Vishwanath S, Carnell GW, Chan ACY, Heeney JL. Immune imprinting and next-generation coronavirus vaccines. Nat Microbiol 2023; 8:1971–85.

50. Kotaki R, Moriyama S, Oishi S, Onodera T, Adachi Y, Sasaki E, Ishino K, Morikawa M, Takei H, Takahashi H, et al. Repeated Omicron exposures redirect SARS-CoV-2–specific memory B cell evolution toward the latest variants. Sci Transl Med 2024; 16.

51. Cao Y, Jian F, Wang J, Yu Y, Song W, Yisimayi A, Wang J, An R, Chen X, Zhang N, et al. Imprinted SARS-CoV-2 humoral immunity induces convergent Omicron RBD evolution. Nature 2022;

52. Park Y-J, Pinto D, Walls AC, Liu Z, De Marco A, Benigni F, Zatta F, Silacci-Fregni C, Bassi J, Sprouse KR, et al. Imprinted antibody responses against SARS-CoV-2 Omicron sublineages. Science (1979) 2022; 378:619–27.

53. Kotaki R, Adachi Y, Moriyama S, Onodera T, Fukushi S, Nagakura T, Tonouchi K, Terahara K, Sun L, Takano T, et al. SARS-CoV-2 Omicron-neutralizing memory B cells are elicited by two doses of BNT162b2 mRNA vaccine. Sci Immunol 2022; 7.
